# Comparative Genomics Reveal a Flagellar System, a Type VI Secretion System and Plant Growth-Promoting Gene Clusters Unique to the Endophytic Bacterium *Kosakonia radicincitans*

**DOI:** 10.3389/fmicb.2018.01997

**Published:** 2018-08-30

**Authors:** Matthias Becker, Sascha Patz, Yvonne Becker, Beatrice Berger, Mario Drungowski, Boyke Bunk, Jörg Overmann, Cathrin Spröer, Jochen Reetz, Gylaine V. Tchuisseu Tchakounte, Silke Ruppel

**Affiliations:** ^1^Leibniz Institute of Vegetable and Ornamental Crops, Grossbeeren, Germany; ^2^Algorithms in Bioinformatics, Center for Bioinformatics, University of Tübingen, Tübingen, Germany; ^3^Institute for Epidemiology and Pathogen Diagnostics, Julius Kühn-Institute–Federal Research Centre for Cultivated Plants, Braunschweig, Germany; ^4^Institute for National and International Plant Health, Julius Kühn-Institute–Federal Research Centre for Cultivated Plants, Braunschweig, Germany; ^5^Leibniz Institute DSMZ-German Collection of Microorganisms and Cell Cultures, Braunschweig, Germany; ^6^Federal Institute for Risk Assessment, Berlin, Germany

**Keywords:** *Kosakonia radicincitans*, Enterobacteriaceae, PGPB, endophytes, comparative genomics, flagellar system, type VI secretion system, plasmid

## Abstract

The recent worldwide discovery of plant growth-promoting (PGP) *Kosakonia radicincitans* in a large variety of crop plants suggests that this species confers significant influence on plants, both in terms of yield increase and product quality improvement. We provide a comparative genome analysis which helps to unravel the genetic basis for *K. radicincitans'* motility, competitiveness and plant growth-promoting capacities. We discovered that *K. radicincitans* carries multiple copies of complex gene clusters, among them two flagellar systems and three type VI secretion systems (T6SSs). We speculate that host invasion may be facilitated by different flagella, and bacterial competitor suppression by effector proteins ejected via T6SSs. We found a large plasmid in *K. radicincitans* DSM 16656^T^, the species type strain, that confers the potential to exploit plant-derived carbon sources. We propose that multiple copies of complex gene clusters in *K. radicincitans* are metabolically expensive but provide competitive advantage over other bacterial strains in nutrient-rich environments. The comparison of the DSM 16656^T^ genome to genomes of other genera of enteric plant growth-promoting bacteria (PGPB) exhibits traits unique to DSM 16656^T^ and *K. radicincitans*, respectively, and traits shared between genera. We used the output of the *in silico* analysis for predicting the purpose of genomic features unique to *K. radicincitans* and performed microarray, PhyloChip, and microscopical analyses to gain deeper insight into the interaction of DSM 16656^T^, plants and associated microbiota. The comparative genome analysis will facilitate the future search for promising candidates of PGPB for sustainable crop production.

## Introduction

Among the approaches to achieve food security and the sustainable handling of resources, one of the most promising is considered the tailored application of microorganism-based inoculants in agriculture, with an expected compound annual growth rate of 9% during 2015–2020 on a global scale (http://www.researchandmarkets.com/reports/3786558). Plant-associated microorganisms have proven to protect their hosts from pathogens and herbivores, thus providing efficient supplements of, or alternatives to pesticides, while other microorganisms were shown to increase nutrient availability, thus promoting plant growth and yield. Over the past two decades effort has been made to explore the association between so-called plant growth-promoting bacteria (PGPB) and non-leguminous plants for producing agricultural inoculants made from bacteria. Being the three most produced cereals of the world and accounting for approximately two-thirds of human food consumption, maize, rice and wheat are of particular interest for the application of PGPB. In contrast to symbiotic, root nodule-colonizing rhizobia (= plant development-enhancing strains of family Rhizobiaceae) and filamentous Actinobacteria from genus *Frankia*, many enteric PGPB, particularly strains from genera *Enterobacter, Klebsiella* and *Kosakonia*, are able to colonize a wide range of hosts. The identification of PGPB is challenging and time-consuming. Reducing the entire microbiota of plants to a few promising candidates with high PGP potential requires numerous experiments on beneficial traits. In 1986 we isolated nearly a thousand bacterial strains from the rhizosphere and phyllosphere of winter wheat (*Triticum aestivum* cv. “Alcedo”) and selected putatively plant growth-promoting strains. One of them possessed stable and high plant growth-promoting as well as yield-promoting activity (Ruppel, 1988). The bacterial strain was explored as the new species *Enterobacter radicincitans*, strain D5/23^T^ (Kampfer et al., 2005), today deposited as type strain at the Leibniz Institute DSMZ-German Collection of Microorganisms and Cell Cultures under DSM 16656^T^. In 2013, the year after we published a draft genome of *E. radicincitans* (Witzel et al., 2012), *Enterobacter* was taxonomically divided into five genera, one of which was named *Kosakonia* (Brady et al., 2013). In the course of this reclassification *E. radicincitans* was renamed *Kosakonia radicincitans*.

Remus (Remus et al., 2000) found that inoculating *K. radicincitans* into its native host, the wheat cv. “Alcedo,” resulted in higher grain yields, indicating that even plants naturally hosting PGPB can show better performance when particular microorganisms are enriched. However, *K. radicincitans* does not only positively affect cereals but also vegetables, for instance of the family *Brassicaceae*, e.g., *Brassica oleracea* (Ruppel et al., 2006), *Raphanus sativus* (Berger et al., 2015) and *Solanaceae*, e.g., *Solanum lycopersicum* (Berger et al., 2013). The PGP capacity of DSM 16656^T^ and other *Kosakonia* strains was experimentally demonstrated. Distinct increases in growth, yield and product quality by *K. radicincitans* DSM 16656^T^ have been confirmed in glasshouse experiments as well as in field trials (Schreiner et al., 2009; Berger et al., 2017, 2018) and highlight the potential of this strain for different cultivation management systems.

PGP *Kosakonia* strains have been found to naturally colonize plants in nearly all major clades of angiosperms. In contrast to root nodule-inducing bacteria (*Frankia* and rhizobia), *Kosakonia* is not restricted to a subgroup of rosids, but does also inhabit asterids and monocots. Among the natural hosts of *K. radicincitans* are many crops of global economic relevance. Most notably are the staple crops maize, rice, wheat and sweet potato, the multipurpose crop sugarcane, the fiber crop cotton, and the biofuel plant *Jatropha*. However, peanut, pineapple, tomato and the beverage plant yerba mate are also among the domesticated plants that host *Kosakonia* (for references see Supplementary Table S1). Most of these *Kosakonia* strains were shown to promote plant growth. To date, full genomes or whole genome shotgun (WGS) sequences of 20 strains of *Kosakonia* have been made available at NCBI (although two of them are also referred to as *Enterobacter*): *K. radicincitans* (DSM 16656^T^, YD4, GXGL-4A, UMEnt01/12), *K. oryzae* (Ola-51, KO348, D4, CGMCC 1.7012), *K. sacchari* (SP1, BO-1, CGMCC 1.12102), *K. arachidis* (Ah-143), *K. pseudosacchari* (JM-387), *K. oryziphila* (REICA_142), *K. oryzendophytica* (REICA_082, LMG 26432), *K. cowanii* (888-76, DSM 18146), *Enterobacter* sp. (R4-368, FY-07).

The recently sequenced and published genomes of *Kosakonia* spp. provided the opportunity to perform comparative genome analyses among *Kosakonia* and between main groups of enteric PGPB. For this purpose we re-sequenced the genome of DSM 16656^T^, the type strain of *K. radicincitans*, using the SMRT (single molecule real time) technology of Pacific Biosciences (PacBio) and applied it as reference. One aim of this study was to evaluate the usefulness of comparative genome analyses for *in silico* prediction of promising bacterial features. Very recently, a comprehensive comparative genome analysis by Levy and co-workers pursued the same goal and found that plant-associated bacteria from across the bacterial kingdom share particular genomic features (Levy et al., 2018).

Here, we present a comprehensive overview of the enteric bacterium *Kosakonia radicincitans*, its phylogenetic relationships and hosts based on the literature. We provide the complete genome sequence and annotation for type strain *K. radicincitans* DSM 16656^T^. Using comparative genomics we were able to unravel the genetic basis for the high PGP competence, motility and competitiveness of this strain. Based on genomic features unique to *K. radicincitans* we developed hypotheses that we tested by *in vivo* experiments applying transcriptomic, bacterial community composition, and microscopical analyses.

## Materials and methods

Details such as primer sequences, links to webpages and applied computer programs are provided in the Supplementary File.

### Pacbio library preparation and sequencing

SMRTbell™ template library was prepared according to the instructions from Pacific Biosciences, following the Procedure & Checklist − 20 kb Template Preparation using BluePippin™ Size-Selection System. Briefly, for preparation of 15 kb libraries 8 μg genomic DNA libraries was sheared using g-tubes™ from Covaris, according to the manufacturer's instructions. DNA was end-repaired and ligated overnight to hairpin adapters applying components from the DNA/Polymerase Binding Kit P6 from Pacific Biosciences. Reactions were carried out according to the manufacturer's instructions. BluePippin™ Size-Selection to 7 kb was performed according to the manufacturer's instructions (Sage Science). Conditions for annealing of sequencing primers and binding of polymerase to purified SMRTbell™ template were assessed with the Calculator in RS Remote, Pacific Biosciences. SMRT sequencing was carried out on the PacBio *RSII* (Pacific Biosciences) taking one 240-min movie for each SMRT cell. In total 1 SMRT cell was run. 98,548 reads with a mean read length of 12,899 bp were obtained.

### Genome assembly, error correction, and annotation

SMRT Cell data was assembled using the “RS_HGAP_Assembly.3” protocol included in SMRT Portal version 2.3.0 using default parameters. The assembly revealed a circular chromosome and two plasmids. Validity of the assembly was checked using the “RS_Bridgemapper.1” protocol. Each genome was error-corrected by a mapping of Illumina reads onto finished genomes using BWA (Li and Durbin, 2009) with subsequent variant and consensus calling using VarScan (Koboldt et al., 2012). A consensus concordance of QV60 could be confirmed for the genome. Finally, an annotation was generated using the software tool for rapid prokaryotic genome annotation (PROKKA) version 1.8 (Seemann, 2014). The genome was deposited in NCBI GenBank under the accession numbers CP018016.1 (chromosome), CP018017.1 (large plasmid), CP018018.1 (small plasmid).

### Gene annotation and functional annotation

Genome annotation was derived from PROKKA, RNAmmer and ARAGORN. Functional annotation was received from the SEED-based “Rapid Annotations using Subsystems Technology” (RAST) tool from the RAST server and pathway classification from “KEGG Orthology And Links Annotation” tool (BlastKOALA).

### Phylogenetic analysis

16S rRNA gene sequences and metadata of published *K. radicincitans* strains, closely related *Kosakonia* species and other genera of enteric PGPB were collected from NCBI databases. The 16S rRNA gene sequences originating from the species whole genome sequence were aligned with MUSCLE and the resulting tree was inferred based on the Maximum Likelihood approach considering the Jukes Cantor model of evolution. An initial tree was obtained by the Neighbor Joining algorithm. Bootstraps values indicated as numbers in percentage close to the nodes represent the confidence level of a clade by resampling the dataset 1,000 times. All algorithms were executed with Geneious (8.1.9). Likewise, a protein tree was built on concatenated amino acid sequences of the phylogenetic marker genes *atpD, gyrB, infB*, and *recA* taking the BLOSUM62 substitution matrix into account. According to the phylogenetic distances and the pairwise identity, received by the BLAST results distinct phylogenetic groups could be determined.

### Genome comparison

The nucleotide and amino acid sequences for the whole genomes were retrieved from NCBI. We compared the DSM 16656^T^ genome sequence to 31 closely related and three distantly related, fully sequenced and annotated genomes of the following bacteria: *K. radicincitans* group (Ola 51^T^, GXGL-4A, YD4, UMEnt01/12, REICA_142), *K. sacchari* group (SP1^T^, R4-368, KO348, BO-1), *K. variicola* group (DX120E, DSM 15968, 342, D5A), *Enterobacter* group (REICA_082, FY-07, SBP-8, P101, DC1, DC3, DC4, ENHKU01, SST3, GS1, 638, UCD-UG_FMILLET), *Citrobacter* (FDAARGOS_122, FDAARGOS_156, FDAARGOS_164, FDAARGOS_165, Y19, CAV1321), *Pseudomonas fluorescens* (F113), *Rhizobium leguminosarum bv. viciae* (3841), and *Bacillus velezensis* (FZB42). To obtain conserved genomic regions a MAUVE (version 2.4.0) alignment (http://darlinglab.org/mauve/mauve.html) was generated for each phylogenetic group: *K. radicincita*ns (KORA), *K. sacchari* (KOSA), *Enterobacter* (ENTERO) and *Klebsiella variicola* (KLEVA) with DSM 16656^T^ as reference. To detect orthologous genes and gene clusters an in-house pipeline was applied. In its first step all proteins were aligned to each other using BLASTp+ by excluding hits with an identity lower 35%, a coverage lower 60% and an e-value cut-off with 1e^−5^, and considering the reciprocal best hit algorithm (RBH) and scoring, as well as the conserved regions. In a second step the orthologous genes/clusters were weighted for their affiliation to a conserved region defined by MAUVE, as mentioned above. In a third step the resulting clusters were divided into the different phylogenetic group clusters for core genome assignment accordingly to their binary absence/presence pattern. To expose differences in specific functional genes or gene clusters occurrences their copy number (for a single gene) or total gene content was extracted from the RBH-table and verified by manual revision of all considered genomes. Finally for visualization SVG files were generated with BRIG (Version 0.95, http://brig.sourceforge.net/) and in-house scripts.

For core genome analysis we chose three representative strains per group [(i) KORA (DSM 16656^T^, YD4, Ola 51), (ii) KOSA (KO348, SP1, BO-1), (iii) ENTERO (DC1, SST3, P101), and (iv) KLEVA (342, DX120E, DSM 15968)] according to the following criteria: (i) endophytic, plant growth-promoting and/or diazotrophic (N_2_-fixing), (ii) isolated from crop plants, (iii) collected in different countries, and (iv) high genetic identity among representatives of each group (Supplementary Table S1).

### Root exudate collection

Tomato plants, *Solanum lycopersicum* cv. “Vanessa” (HILD) were cultivated in a sand: vermiculite mixture (v: v, 1:1) for 4 weeks. For exudate collection the substrate was gently removed by washing the roots. Then, the tomato plants were kept in sterile bi-distilled water for 1 h, followed by a 4 h collection period in bi-distilled water. The root exudate-containing water was two step-vacuum filtered (through 0.45 and 0.2 μm sterile filters) and then frozen and freeze-dried for further analysis. The C/N ratio was measured using a CNS Vario EL analyzer (Elementar).

### Customized microarray: cultivation of bacteria, microarray design, analysis and quality control

*K. radicincitans* DSM 16656^T^ was grown in 20 mL shaking culture at 28°C and 190 rpm for 24 h in mineral medium (Gerhardt et al., 1994) supplemented either with sucrose and glucose or with tomato root exudates (filter sterilized), both adjusted to 5 g C L^−1^. After 24 h bacteria were pelleted and frozen in liquid nitrogen. RNA was extracted from the pellet using innuPREP RNA Mini Kit (Analytik Jena) and quantified by electrophoresis using an Agilent 2100 Bioanalyzer®. Four replicates of each bacterial treatment were fragmented, labeled and hybridized to the DSM 16656^T^ custom array. Using the web based eArray application (Agilent Technologies) we designed a microarray on an 8 × 60 k formatted slide using 5,512 protein coding genes of *K. radicincitans* DSM 16656^T^, which could be assigned unambiguously to PacBio sequences. For each gene 10 specific oligonucleotide probe sequences were designed for hypervariable regions and spotted along with 1,319 internal Agilent quality controls. Raw signal intensity values were determined using Agilent standard protocol for Affymetrix Microarray Analyzer from ATLAS-Biolabs. Probe level values (Agilent “gProcessedSignal”) were preprocessed using Quantile normalization from R-package preprocessCore and log transformed. The Agilent “IfPosAndSig” values 1 or 0 were used as detection-threshold. In order to estimate the quality of the used samples, the Pearson correlation between all sample pairs was calculated and used as distance measurement. Using Pearson correlation together with “Ward” linkage method in the hierarchical clustering one sample was detected to be an outlier and removed. Follow-up analyses were performed on the seven remaining samples. In order to determine genomic regions of *K. radicincitans* DSM 16656^T^ differentially expressed (up- or down-regulated), a genome-wide sliding window calculation was performed considering a window size of 15 consecutive genes.

### Microbiome phylochip analysis

*K. radicincitans* DSM 16656^T^ was grown, washed and diluted as described in Ruppel et al. (2006). Seeds of tomato, *S. lycopersicum* cv. “Vanessa” were either exposed to 10^8^ cells of DSM 16656^T^ or to pure buffer solution (0.05 M NaCl = control), then germinated and again inoculated, this time by spraying 10^8^ cells onto the leaves of each plant at seedling (2-leaf) stage. Pure buffer was sprayed on controls. Plants were grown in non-sterile quartz sand (grain size 0.5–1.0 mm). Two weeks after spraying, leaves and roots were collected separately in liquid nitrogen, freeze-dried and total DNA was extracted using DNeasy Plant MiniKit (Qiagen). Colonization with *K. radicincitans* was quantified using a *K. radicincitans*-specific TaqMan™ probe labeled with 6-FAM and a black hole quencher at the 3′-end was used in quantitative real-time PCR using the plant TEF gene as a reference gene as described in Ruppel et al. (2006). The absolute bacterial cell quantification was conducted using the target gene calibration curve ranging from 10 to 10^9^ copy numbers per μL. In high quality DNA the bacterial 16S rRNA genes were amplified. Sixteen samples (four replicates of root and shoot non-inoculated and inoculated by *K. radicincitans*) were moved forward for hybridization at Second Genome, Inc. PhyloChip Control Mix™ was added to each amplified product. Labeled bacterial products were fragmented, biotin labeled and hybridized to the PhyloChip™ Array, version G3 at Second Genome, Inc. Each scan was captured using standard Affymetrix software (GeneChip® Microarray Analysis Suite). Data analysis was performed using Second Genome's PhyCA-Stats™ Analysis Software (http://www.secondgenome.com/platform/services/phylochip/).

### CLSM and TEM microscopy

*S. lycopersicum* cv. “Micro-Tom” were raised as *in vitro* cultures. Young seedlings were inoculated with GFP-labeled *K. radicincitans* as described in Witzel et al. (2017). Bacterial root colonization was recorded with a Zeiss LSM 510 META laser scanning confocal microscope (Carl Zeiss Jena GmbH). Bacterial eGFP fluorescence signals were captured using argon laser excitation at 488 nm (BP505-550 180 filter, Plan Apo 63/1.4 oil lens), and root images were captured using bright field settings.

Conventional negative contrast staining was used for electron microscopic investigations. Cells of *K. radicincitans* DSM 16656^T^ were taken from semi solid (0.5%) agar and placed in 0.05 M saline solution. One drop of this bacterial solution was applied to Pioloform-carbon-coated, 400-mesh copper grids (Plano GmbH) for 10 min, fixed with 2.5% aqueous glutaraldehyde solution for 1 min, stained with 2.5% uranyl acetate solution for 1 min, and examined by transmission electron microscopy using a JEM-1400 Plus (JEOL) at an acceleration voltage of 120 kV.

## Results

### The genome annotation of DSM 16656^T^ revealed one chromosome, two plasmids and a high percentage of genes involved in plant growth-promotion

*Kosakonia radicincitans* type strain DSM 16656^T^ is an enteric bacterium that was shown to promote plant growth (Supplementary Table S2). Exhibiting two scaffolds and no plasmid sequence the preexisting draft genome of DSM 16656^T^ was incomplete. Here we provide the fully sequenced and annotated genome, one chromosome (5,817,639 bp) and two plasmids (290,108 bp and 14,706 bp) of DSM 16656^T^ (GenBank: CP018016.1, CP018017.1, CP018018.1) generated by combined PacBio and Illumina HiSeq sequencing. Apart from a rather small fraction of 6.3% (= 370 genes) biological functions could be assigned to all remaining of the 5,839 coding sequences (CDS) of DSM 16656^T^ combining results from BlastKOALA (KEGG), Interpro (GO, protein domains) and PROKKA (note that there are only 5,827 genes and 5,712 CDS in the NCBI annotation; (Supplementary Table S3). Almost one third (32.9% = 1,800 genes) of all annotated CDS of DSM 16656^T^ are involved in environmental information processing, 288 genes (= 5.3%) code for secretion systems and 214 genes (= 3.9%) for proteins involved in cell motility. A category summarizing PGP genes does not exist in the applied functional classification tools (RAST from SEED and BlastKOALA from KEGG), but summing up the CDS allocated to PGP we found 243 genes (= 4.4% of the genome) of *K. radicincitans* DSM 16656^T^ (Table 1). Throughout the article we compare functional classification and genomic composition of *K. radicincitans* to three other groups of enteric PGPB in order to distinguish shared features from unique features of the *K. radicincitans* species and the type strain DSM 16656. The following paragraph describes how these groups have been defined.

**Table 1:** Genes of *Kosakonia radicincitans* DSM 16656^T^ involved in plant growth-promotion according to literature on homologous genes of other bacteria.

**Symbol**	**Traits**	**Genes**
a	Nitrogen fixation and metabolism	*amtB*; *anfA*, anfD, anfF^*^, anfGH, anfK, anfO; *narB, narG*(2), nar*HIJKL, narX*; *nasR, nasD*; *nifAB, nifDEF, nifHIJKLMN, nifQ, nifSTUVWX, nifZ*; *nirB, nirD*; *nrtABC*
b	Phosphate solubilization	*phoAB, phoC*(2) *phoE, phoH, phoR, phoU; pqqE*-like*; pstABC, pstS*(2)
c	IAA production	*ipdC*
d	GABA production	*gabD*(3), *puuE*
e	ACC deaminase	*dcyD*
f	Acetoin and butanodiol synthersis	*als*; *budAB, budC*(2); *poxB*
g	H_2_S production	*cysCD, cysG*(2), *cysHI, cysJ*(2), *cysN*
h	Trehalose metabolism	*sugBC*; *otsAB*; *treA*(2), *treB, treRS, treYZ*
i	Catalase	*katB, katE, katG, katN*
j	Peroxidases	Peroxidase gene homolog (34)
k	Superoxide dismutase	*sodABC*
l	Quorum sensing	*lsrB*(2), *IsrD, IsrFG, IsrK; luxS*(2)
m	Heat shock proteins	*dnaJ, dnaK*(2); *groE*
n	Cold shock proteins	*cspA*(5), *cspE, cspG, cspJ*
o	Glycine-betaine production	*opuAB, opuAC, opuCA*(2), *opuCB*(4), *opuCC*(2); *osmY*(2); *proV*(2), *proWX*
p	Effective rhizosphere colonizer	*xerC*(6)
q	Siderophore production	*acrB*(3); *besA*; *btr*(3); *efeB, efeO, efeU*; *entA*(2), *entBC, entEF, entH, entS*(2); *exbB*(3), *exbD*(3); *feoABC*; *fepA*(2), *fepB, fepDE, fepG*; *fes*; *fhuA*(3), *fhuB*(3), *fhuC*(3), *fhuD*(3), *fhu*EF; *fptA*; *mbtH*-like; *mdtA*(2), *mdtB*(2), *mdtC*(2); *mexA*(2), *mexB*; *tonB*; *yusV*(2)
r	Pyocin	*prtN*
s	Phenazone production	*yddE*(2)
t	4-hydroxybenzoate production	*ubiC*
u	Chitinase production	*chiA1*(2), Chitinase gene homolog(2)

* *Gene within the anf gene cluster encoding a flavin nucleotide-binding protein similar to flavotoxin-encoding nifF*.

### Phylogenetic studies allowed a preselection of other enteric PGPB for comparative analyses

**Figure 1:**
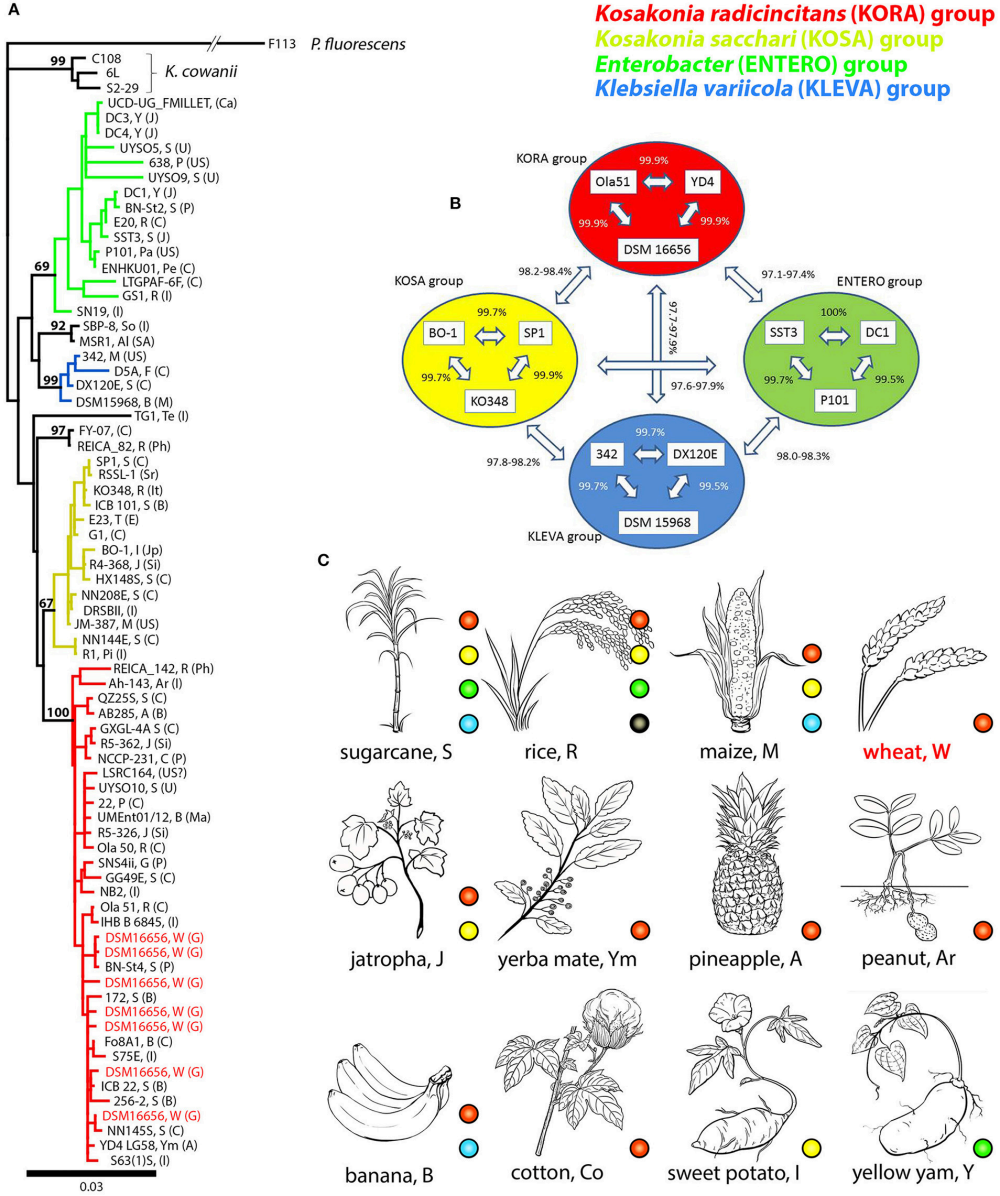
Phylogeny, selection of reference strains and plant hosts of PGP *Kosakonia* and closely related bacterial species; Four major groups are considered and depicted in different colors: red, *Kosakonia radicincitans* (KORA) group; yellow, *Kosakonia sacchari* (KOSA) group; green, *Enterobacter* (ENTERO) group; blue, *Klebsiella variicola* (KLEVA) group. Capital letters in **(A,C)** refer to plant hosts (Supplementary Table S1). **(A)** 16S rRNA gene tree of *Kosakonia* strains and a selection of *Enterobacter* and *Klebsiella* strains closely related to *Kosakonia*. Outgroup is *Pseudomonas fluorescens*. Three representatives of putatively pathogenic *Kosakonia cowanii* are shown. All 16S rRNA gene copies of DSM16656^T^ (= type strain of *Kosakonia radicincitans*) are considered, but only a single (the most prominent) copy of other strains. Capital letters in brackets represent sampling site countries (Supplementary Table S1). **(B)** Selection of 12 bacterial strains from gene tree shown in **(A)**; **(C)** Important crop plants hosting enteric PGPB depicted in **(A)**; only hosts of published strains are shown. Black dots and branches represent SBP-8, MSR1, TG1, and REICA-082, the strains nested in between the four depicted major groups of *Kosakonia*/*Enterobacter*/*Klebsiella*.

Multiple copies of 16S rRNA genes were found in fully sequenced *K. radicincitans* and closely related *K. oryzae* strains: DSM 16656^T^ (seven copies), Ola 51^T^ (five copies), GXGL-4A (seven copies), YD4 (seven copies), UMEnt01/12 (two copies). The 16S rRNA “main” (or consensus) gene sequence—represented by the most identical or very similar copies (Supplementary Figure S1A)—was chosen for phylogenetic analyses. A phylogeny of *Kosakonia* and closely related strains was based on these main sequences; only for *K. radicincitans* DSM 16656^T^ all copies have been considered (Figure 1A). Our phylogenetic analyses of 16S rRNA gene sequences based on 70 bacterial strains reveal the presence of two distinct clades of PGP *Kosakonia*. The first (Figure 1A, depicted in red) comprises *K. radicincitans, K. oryzae, K. oryziphila*, and *K. arachidis*, the second (Figure 1A, depicted in yellow) comprises *K. sacchari, K. oryzae*, and *K. pseudosacchari*. Note that *Kosakonia* strains named *K. oryzae* do not form a monophyletic group. A third *Kosakonia* clade comprises *Enterobacter* sp. FY-07 and *E. oryzendophyticus* REICA 082, more recently named *Kosakonia oryzendophytica*. *Kosakonia cowanii* is outgroup to these three *Kosakonia* groups (Figure 1A). A more comprehensive 16S rRNA gene tree based on 195 bacterial strains (Supplementary Figure S2) and a phylogeny based on concatenated amino acid sequences of reference genes *atpD, gyrB, infB*, and *recA* (Supplementary Figure S3) show similar affiliations. In the following analyses we focus on the two large *Kosakonia* subgroups that were shown to contain many PGPB, and we refer to them as the *K. radicincitans* group (Figure 1, KORA clade, red) and the *K. sacchari* group (Figure 1, KOSA clade, yellow). Sharing >97% 16S rRNA gene sequence identity with *K. radicincitans*, strains of closely related *Klebsiella variicola* and *Enterobacter* sp. (both family *Enterobacteriaceae*) were also included in the analysis. The selected strains were shown to be naturally associated with crop plants (Figure 1C) and capable of promoting plant growth. Here we refer to the *Klebsiella variicola* group (Figure 1, KLEVA clade, blue) and the *Enterobacter* group (Figure 1, ENTERO clade, green). A detailed list of *Kosakonia* strains (including accession, reference, location and 16S rRNA gene sequence identity to DSM 16656^T^) and the selected *Enterobacter* and *Klebsiella* strains is provided in Supplementary Table S1. Additionally, data on a few *Citrobacter* strains of particular interest (s. below) is given in this table.

### Functional classification of four subgroups of enteric PGPB show large fraction of *Kosakonia radicincitans* genes involved in motility and chemotaxis

**Figure 2:**
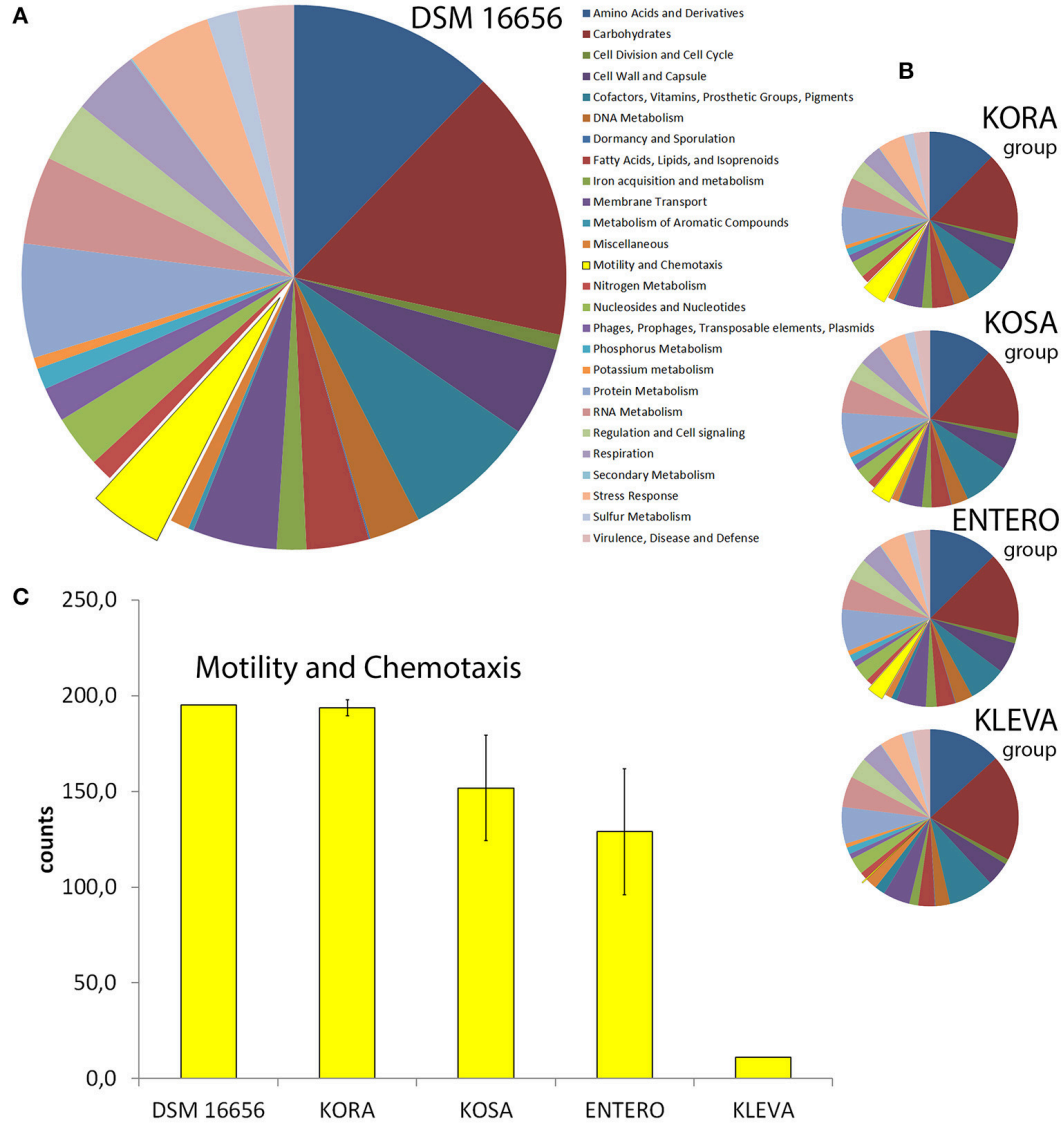
Functional classification of DSM 16656^T^, *Kosakonia radicincitans* (KORA group), *K. sacchari* (KOSA group), *Enterobacter* spp. (ENTERO group) and *Klebsiella variicola* (KLEVA group) by SEED using RAST-annotated genomes. **(A)** DSM 16656, **(B)** each pie chart represents the average of a group of three bacterial strains (KORA, KOSA, ENTERO, and KLEVA group). The size of a section corresponds to the number of counts for the functional category; a single gene may have multiple counts when present in multiple sub-categories; motility and chemotaxis are depicted in yellow. **(C)** Extraction from **(A)** and **(B)**; error bars show standard deviation of the three strains.

Using RAST-annotated genomes we studied the functional classification of DSM 16656^T^ and other enteric PGPB (Figure 2). We compared the four robust groups of enteric PGPB, prior determined by 16S rRNA gene sequence analyses. We chose three representative strains per group [(i) KORA (DSM 16656^T^, YD4, Ola 51), (ii) KOSA (KO348, SP1, BO-1), (iii) ENTERO (DC1, SST3, P101), and (iv) KLEVA (342, DX120E, DSM 15968)]. 16S rRNA gene identities among selected representatives of each clade are 99.5–100% and between clades 97.1–98.4% (Figure 1B). Although the overall pattern of functional classification is very similar in DSM 16656^T^ and the four investigated groups of enteric PGPB (Figures 2A,B), there is one striking difference (highlighted in yellow). The KORA group has more genes for motility and chemotaxis than the three other groups, particularly more than the KLEVA group (Figure 2C). All three *K. radicincitans* strains analyzed have an equally high number of genes involved in motility and chemotaxis, and all three *Klebsiella* strains an equally low number of these genes, but members of the KORA group and the ENTERO group show higher variability in the number of genes allocated to motility and chemotaxis (as visualized by error bars). A detailed genome map of DSM 16656^T^ is provided in Supplementary Figure S4, and the number of genes per functional category for all representative strains is given in Supplementary Figure S5.

### Comparison of core genomes simplifies the search for *Kosakonia*-specific characteristics as well as features shared between enteric PGPB

**Figure 3:**
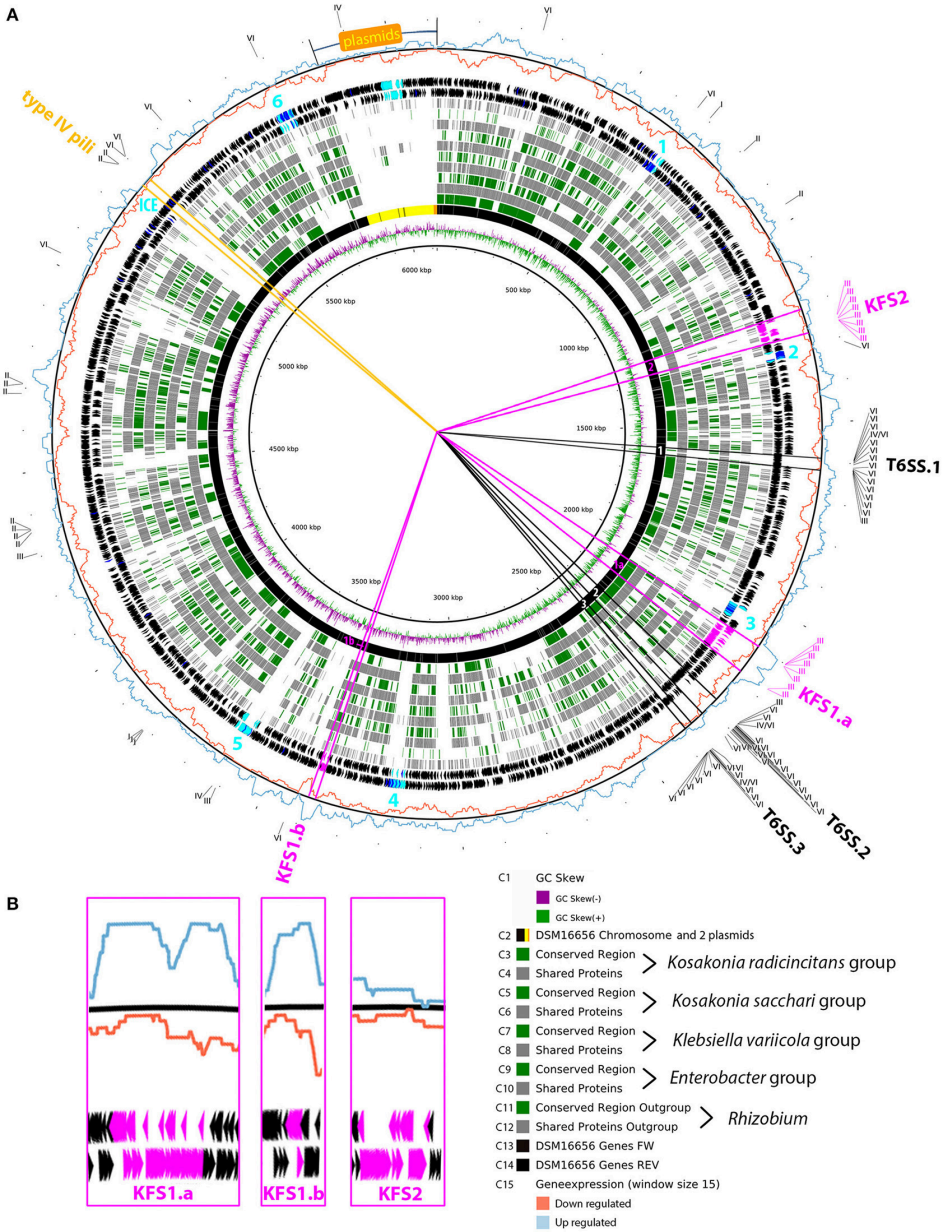
Core genome comparison. **(A)** Comparison of the *Kosakonia radicincitans* DSM 16656^T^ genome (including plasmid sequences highlighted by yellow/orange area in circle C2) to core genomes of other enteric PGPB (KORA, KOSA, ENTERO and KLEVA group). Gray lines and boxes in circles C4, C6, C8, C10, C12 point to the presence of genes (according to NCBI BLASTp searches) shared among the three strains of a core genome; green lines and boxes in circles C3, C5, C7, C9, C11 represent collinear units of nucleotide sequences determined by MAUVE alignments, i.e., conserved gene clusters in which the order of genes is equal to DSM 16656^T^. Light blue genes depicted in circles C13 and C14 belong to six phage regions detected by PHAST software, and are highlighted by light blue numbers 1–6; dark blue genes have phage-related functions according to our own annotation pipeline. The ICE region is an *i*ntegrative *c*onjugative *e*lement. Pink lines demarcate genes of flagellar systems (KFS1 and KFS2), black lines genes of type VI secretion systems (T6SSs), and orange lines genes of type IV pili. Due to the homology of the flagellar basal body and the transmembrane export complex of injectisomes, eight motility genes per flagellar system were annotated to type three secretion systems (T3SSs). The outermost circle shows the differential expression of the transcriptome of DSM 16656^T^ when exposed to tomato root exudates, captured by a sliding window of 15 consecutive genes; blue graph, up-regulated genes; red graph, down-regulated genes. **(B)** Magnification of flagellar system genes depicted in **(A)** highlighting the different expression of KFS1 and KFS2.

Considering the same 12 strains that were used for the abovementioned functional classification we determined the core genome for each group of three enteric PGP strains (KORA, KOSA, ENTERO, KLEVA). We compared these core genomes and the genome of a *Rhizobium* outgroup (*R. leguminosarum bv. viciae* 3841) to DSM 16656^T^ (Figure 3). The purpose of this fast and easy comparison was to simplify the search for (i) genomic features that are shared between DSM 16656^T^ and closely related enteric PGPB, (ii) genomic features that are unique to *Kosakonia radicincitans*, and (iii) genomic features that are unique to DSM 16656^T^. Although *K. radicincitans* strains from Germany (DSM 16656^T^), China (Ola 51^T^) and Argentina (YD4) share 99.9% of their 16S rRNA gene sequence (Figure 1B), genomes of these strains exhibited significant rearrangements, with approximately half of the gene order conserved, as depicted in circle C3 of Figure 3. White areas in Figure 3 ranging centripetally from circle C3 to circle C12 represent genomic regions of DSM 16656^T^ lacking in all four core genomes and *Rhizobium*. Seven of these regions are mobile genetic elements (MGEs), of which six have been identified as phage regions by PHAST software (Zhou et al., 2011; light blue numbers in Figure 3A), and another region is dominated by genes defining integrative conjugative elements (ICEs). One of the MGEs, phage region 2, consisting of 21 phage and six hypothetical genes, is different since occurring in both *Kosakonia* core genomes (KORA and KOSA), and its gene order is conserved among the three strains of *K. radicincitans* (Figure 3A, circles C3 and C5), which may indicate an important function.

### Species-specific genomic features: *K. radicincitans* has two flagellar systems and three type VI secretion systems

The genome of DSM 16656^T^ carries genes of five types of secretion systems (I, II, III, IV, and VI), particularly of type III (T3SS) and VI (T6SS), indicating that *K. radicincitans* has evolved an arsenal of gene systems for environmental interaction. Whereas, T6SSs encode injectisomes, which export effector proteins to the extracellular milieu, eukaryotic cells or other bacteria, genes allocated to T3SS are not necessarily coding for injectisomes, but may contribute to flagella biosynthesis. In *K. radicincitans* DSM 16656^T^ genes of T3SS cluster together with chemotaxis genes and all other genes required for flagella biosynthesis, suggesting that this strain uses “T3SS” genes for motility purposes. Genomic regions encoding important secretion systems are highlighted in Figure 3. The core genome comparison nicely illustrates that the KORA group — but none of the other groups — possess the second flagellar system (KFS2, *Kosakonia* Flagellar System 2). KFS2 of strain DSM 16656^T^ is a compact gene cluster comprising — apart from five genes — only essential flagellar genes; the other flagellar system (KFS1) is divided into two gene clusters, KFS1.a and KFS1.b, with 1,148 genes in between. The comparison of core genomes (Figure 3A) shows that most genes of KFS1.a and KFS1.b are shared between the KORA, the KOSA and the ENTERO group, but missing in the KLEVA group, an observation that matches common knowledge that *Klebsiella* is a non-motile bacterium.

The order of the 71 genes constituting KFS1.a is highly conserved in *K. radicincitans*, but the order of 55 genes constituting KFS2 varies between strains of this species (Figure 3A, circle C3) probably indicating that KFS2 is rapidly evolving in *K. radicincitans*. The conserved Flg22 motif of the flagellin molecule, and particularly its C-terminal Flg15 segment, may induce MAMP (microbe-associated molecular pattern) triggered immunity. The Flg15 peptide sequence of *K. radicincitans* KFS1 and *E. coli* are identical and differ from *Pseudomonas aeruginosa* in two of the four C-terminal residues (Supplementary Figure S6). The Flg15 peptide sequence of *K. radicincitans* KFS2 has an additional AA substitution at the very last position of the C terminus.

Next to KFS2 there is another duplicated secretion system in the KORA group, which is missing in other enteric bacteria: an additional type VI secretion system (T6SS.3). The two T6SS clusters commonly found in *Enterobacter*, also occur in *K. radicincitans*, here called T6SS.1 and T6SS.2. However, the T6SS.3 of the KORA group is new to enteric bacteria. Genes of T6SS.3 are completely missing in the KOSA, ENTERO and KLEVA group and also in the *Rhizobium* outgroup. The order of T6SS.3 genes in the KORA group is highly conserved (Figure 3).

### Pairwise genome comparisons confirmed assumptions based on core genome analysis

**Figure 4:**
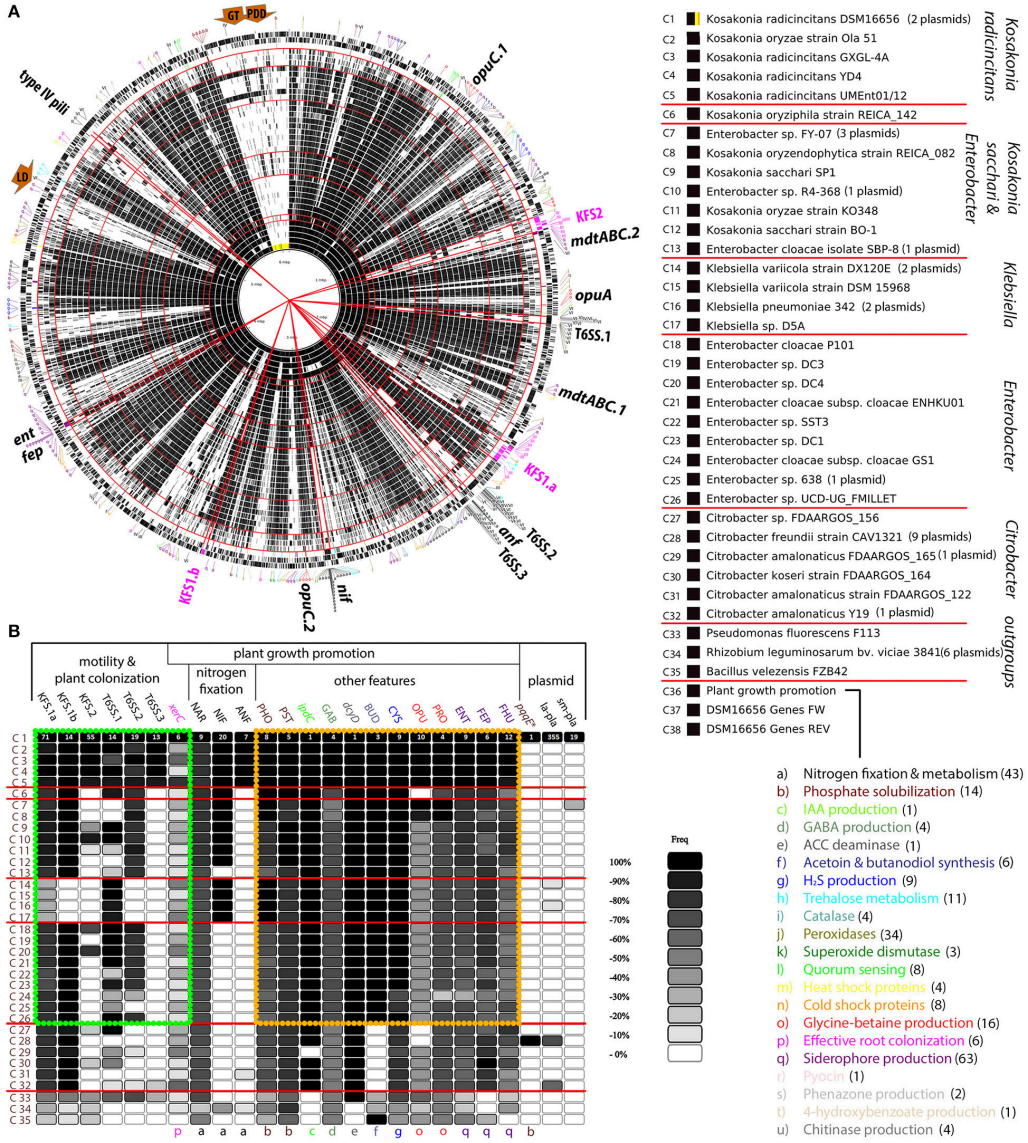
Genome comparison of *Kosakonia radicincitans* DSM 16656^T^ to 31 other strains of enteric bacteria and three strains of non-enteric bacteria. **(A)** Comparative genome map including plasmid sequences (highlighted by yellow and orange area in circle C1). Circles C1 to C35 depict each one bacterial strain; black lines within a circle represent genes. The order of strains is given by 16S rRNA gene sequence identities to DSM 16656^T^, descending from highest to lowest. Circle C36 depicts genes supposed to be involved in plant growth-promotion (PGP); lowercase letters in outermost circle refer to particular PGP functions (Table 1). Red circular lines separate phylogenetic groups. Red straight lines demarcate gene clusters of particular interest, such as PGP genes, type VI secretion systems, type IV pili, and flagellar systems. **(B)** Selection from **(A)** depicting important loci and gene clusters with significant impact on plant growth and plant colonization. Each box represents a gene count per locus for a particular strain compared to DSM 16656^T^. A locus may consist of 1–71 coding sequences in DSM 16656^T^ or entire plasmids with up to 355 coding sequences (la-pla, large plasmid; sm-pla, small plasmid); black boxes represent the maximum count (100%) and white boxes the minimum count (< 10%) of genes found in DSM 16656^T^, gray shadings represent percentages in between. The exact number of genes per box is given in Supplementary Table S5. The yellow rectangle frames some of the most significant PGP genes of *Kosakonia, Enterobacter* and *Klebsiella*, the green rectangle frames genes involved in motility and plant colonization. Dark brown arrows, exploitation of unusual carbon sources (LD, lichenan degradation; GT, glycerol transformation; PDD, 1,2-propanediol degradation).

The comparison of core genomes of PGPB was helpful for rapidly distinguishing genes that DSM16656^T^ shares with different groups of enteric bacteria and genes putatively unique to the KORA group, but whether these candidate genes are indeed restricted to *K. radicincitans* had to be confirmed by a thorough analysis applying a larger number of bacterial genomes (Figure 4A). We compared the DSM 16656^T^ genome sequence to 31 closely related and three distantly related, fully sequenced and annotated bacterial genomes listed in the Material and Method section. From hundreds of *Klebsiella* and *Enterobacter* strains available at NCBI the given ones were chosen due to their endophytic lifestyle and their PGP potential as described in literature (Supplementary Table S1).

The genome comparison of the 35 individual strains of bacteria confirms assumptions based on the core genome analysis: T6SS.3 appears to be restricted to taxa of the KORA group; all five strains of this group included in the genome comparison share the corresponding genes (Figure 4B). BLASTp searches to NCBI databases support this result, yielding very low gene sequence similarities to DSM 16656^T^ of strains other than *K. radicincitans* (average identity for all T6SS.3 genes < 68.5%, Supplementary Table S4).

Similar to the *K. radicincitans*-specific flagellar system (KFS2), T6SS.3 is reduced to the essential core component genes required for T6SS biosynthesis, regulation and functioning (Supplementary Figure S7). The only exception is a gene encoding a T6SS accessory protein of unknown function, here called *tagK1*. While *K. radicincitans* strains share 95% amino acid sequence identity of *tagK1*, the next best hit outside this species shares only 55% sequence identity with DSM 16656^T^ (Supplementary Table S4). The genomic neighborhood of *K. radicincitans* T6SS.3 harbors genes for multiple antibiotic resistance (*marR*), multidrug resistance (*mdtN, emrB*), and resistance to oxidative stress (*marR, katG*; Supplementary Figure S7). Remarkably, *Pseudomonas fluorescens* F113 shares more T6SS.3 genes with *K. radicincitans* than any *Kosakonia sacchari, Klebsiella*, or *Enterobacter* strain included in our comparative genome analysis (Figure 4B).

Just like T6SS.3, KFS2 is shared by all members of the KORA group but in contrast to T6SS.3 it is not absent from all the other groups of enteric bacteria. Two *Enterobacter* strains (P101 and DC4) and one strain from the *K. sacchari* group (R4-368) possess KFS2; *K. oryzae* SP1 has half of KFS2 (Figure 4).

### Shared genomic features: *K. radicincitans* and other species of enteric PGPB share the bulk of genes involved in plant growth-promotion

Apart from flagellar systems and type VI secretion systems our analysis focused on genes reported to be involved in PGP: most notably genes for atmospheric nitrogen fixation, siderophore production, phosphate solubilization and glycine-betaine production. The corresponding genes are listed in Table 1 and depicted by lowercase letters in Figure 4 and Supplementary Figure S4. Important regions of the *K. radicincitans* DSM 16656^T^ genome are indicated by straight red lines and arrows (Figure 4A). The exact numbers of homologous genes illustrated in Figure 4B are given in Supplementary Table S5.

The bulk of genes encoding PGP traits are shared between *Kosakonia* (both large groups), *Enterobacter* and *Klebsiella* (framed by a yellow rectangle in Figure 4B), but a significant fraction of genes required for atmospheric nitrogen fixation and for protecting host plants from osmotic stresses via glycine-betaine (GB) production is unique to *K. radicincitans* or shared with only a subset of other strains. GB production requires the uptake of GB precursor compounds via osmoprotectant uptake systems (Opu). There are three *opu*-gene clusters in *K. radicincitans* (Figure 4): *opuC.1* is shared with the *K. sacchari* group and the *Klebsiella* group but missing in most strains of *Enterobacter* and *Citrobacter, opuC.2* in contrast is shared with *Enterobacter* and *Citrobacter* but missing in the *K. sacchari* and the *Klebsiella* group, and *opuA* is restricted to *K. radicincitans* and *Rhizobium*.

Although atmospheric nitrogen fixation is an extremely energy-consuming process, *K. radicincitans* has two gene clusters for this purpose, the *nif* regulon and *anf* operon. The *nif* regulon is shared with taxa from the *K. sacchari* group, the *K. variicola* group and *Rhizobium*, but missing in taxa from the *Enterobacter* group (Figure 4). The *anf* operon is not found in any other species included in the analysis but is known from distantly related taxa.

Next to atmospheric nitrogen fixation, phosphate solubilizing activity is supposed to be one of the major advantages provided by PGPB. Most genes for phosphorus assimilation (*pho, pst*) and siderophore production/transport are shared among enteric PGPB (Figure 4). However, while *K. radicincitans* shares the largest gene cluster for siderophore production (*ent*/*fep*) and an *mdtABC* operon (*mdtABC*.1) with almost all other strains included in the analysis, other genes for siderophore production are unique to *K. radicincitans*, such as a duplicated *mdtABC* operon (*mdtABC*.2) next to KFS.2 (Figure 4).

Taken together, *K. radicincitans* DSM 16656^T^ is well equipped with genes attributed to PGP and differs from many other enteric bacteria predominantly by the presence of its gene clusters (*nif* and particularly *anf*) for atmospheric nitrogen fixation.

### Strain-specific genomic features of *K. radicincitans* DSM 16656^T^: most large plasmid genes are shared with *Citrobacter* but absent from other *Kosakonia* strains

**Figure 5:**
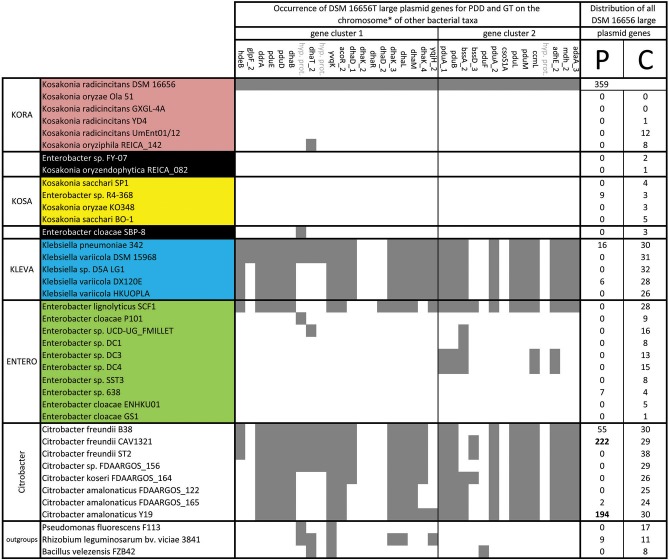
Occurrence and distribution of large plasmid genes of *K. radicincitans* DSM 16656^T^ in chromosomes and plasmids of other bacterial taxa; GT, glycerol transformation; PDD, 1,2-propanediol degradation; P, plasmid; C, chromosome; *none of these PDD and GT genes occur on plasmids in the other investigated taxa.

Our new genome sequencing approach of *K. radicincitans* DSM 16656^T^ revealed the presence of one chromosome and two plasmids (yellow and orange highlighted area in Figures 3A, 4A; for simplification purposes plasmids were included as part of the circular genome map). The comparative genomics approach showed that DSM 16656^T^ shares its small plasmid with the closely related *Enterobacter* sp. FY-07, a cellulose producing strain from China, and its large plasmid with the distantly related *Citrobacter freundii* CAV1321 (USA) and *Citrobacter amalonaticus* Y19 (South Korea; Figures 4B, 5). Genes of CAV1321 and Y19 appeared also as best hits when blasting large plasmid genes of DSM 16656^T^ to NCBI databases. The small plasmid of DSM 16656^T^ carries 19 genes, the large plasmid 359 genes, of which 355 are coding sequences (Supplementary Table S3). A notable fraction (38%) of large plasmid genes are unique to the large plasmid, i.e., missing on the chromosome and the small plasmid of DSM 16656^T^. Among the 57 unique genes of the large plasmid that could be assigned to KEGG pathways 39% refer to microbial metabolism in diverse environments, indicating that the large plasmid increases the capability of DSM 16656^T^ to cope with different habitats (Supplementary Table S6). Remarkably, the large plasmid is missing from all other fully sequenced *Kosakonia* genomes, and from representative *Klebsiella* and *Enterobacter* strains included in the analysis (Figure 4) suggesting that the plasmid may have been transferred from *Citrobacter* to DSM 16656^T^. 145 of the 355 coding sequences of the large plasmid of DSM 16656^T^ are shared exclusively with *Citrobacter* and missing in other *Kosakonia* strains. The contribution of the presumably *Citrobacter*-derived plasmid to the PGP potential of DSM 16656^T^ appears to be low as measured by the usual PGP repertoire (Table 1), since all genes allocated to PGP occur on the chromosome, except a single (*pqqE*-like) gene from the large plasmid (Figure 4B).

### The large plasmid of DSM 16656^T^ carries many genes for exploiting plant-derived carbon sources

Although 14% of all KEGG identified genes of the large plasmid encode ABC transporters for nutrient uptake (e.g., mineral and carbohydrate transporters), this percentage is low compared to chromosomal genes, where 28% code for ABC transporters (Supplementary Table S6). Consistently, a good portion of the ABC transporter spectrum is covered by the chromosome of DSM 16656^T^ and only a single additional gene (*troA*) is contributed by the plasmids. However, the fraction of genes involved in propanoate and glycerolipid metabolism is 5-fold higher in the large plasmid (Supplementary Table S6) indicating that plasmid genes may play a significant role in exploiting plant-derived carbon sources: the presence of *pdu* genes (*pduABDEFLMPQ*) for propanediol degradation (PDD) and *dha* genes (*dhaBDKLMT*) for glycerol transformation (GT) on the large plasmid (Figure 4) suggests that DSM 16656^T^ has the potential to anaerobically degrade rhamnose and fucose in plant cell walls and glycerol in plant lipids. Genes for PDD and GT are shared between DSM 16656^T^, *Citrobacter* and *Klebsiella* (Figure 4A), but are missing from other *Kosakonia* strains and *Enterobacter* (with the exception of *E. lignolyticus* SCF1; Figure 5). Although these genes constitute a prominent feature in *Citrobacter* and *Klebsiella*, they are not located on plasmids of the investigated strains but on chromosomes (Figure 5).

### DSM 16656^T^ carries further genes for exploiting plant-derived carbon sources on the chromosome

Particular genes on the chromosome have further increased the potential of DSM 16656^T^ to exploit plant-derived carbon sources. Genes for degradation of lichenan, a beta-1,3-1,4-glucan from cell walls of lichen and—in a slightly different form—from barley and oat, may be attributed to horizontal gene transfer from *Bacillus* to *Kosakonia*. Three of the genes required for lichenan degradation (*licABR*) are shared between *Bacillus subtilis* FZB42 and two *Kosakonia* strains, DSM 16656^T^ and UMEnt01/12, but are missing in other *Kosakonia, Enterobacter, Klebsiella*, and *Citrobacter* strains.

### The two flagellar systems of *K. radicincitans* DSM 16656^T^ are operating in different environments

We assumed that the existence of two distinct flagellar systems (KFS1 and KFS2) in *K. radicincitans* reflects different purposes, i.e., the individual operation of each flagellar system in different environments. To test this hypothesis we designed a DSM 16656^T^-specific microarray and exposed bacterial cells from liquid culture to root exudates (C/N ratio of 9.39) of tomato, *S. lycopersicum cv*. “Vanessa”. The tomato cultivar “Vanessa” was shown to respond to DSM 16656^T^ by increased growth and yield (Berger et al., 2013, 2017). In this experiment we simulated the condition, in which the flagellum for swimming toward host would be required. Determining the number of differentially expressed genes in a sliding window of 15 consecutive coding sequences (CDS) throughout the genome we searched for highly enriched sets of differentially expressed genes, depicted in the outermost circle of Figure 3. Applying a *p*-value of 0.05 and a FC threshold of 1.5, a fraction of 52% of motility and chemotaxis genes (111 of 214 CDS) was differentially expressed. However, while nearly the same number of all differentially expressed CDS was up- (1,197) and down-regulated (1,135), most of the differentially expressed chemotaxis and motility genes, namely 86%, were up-regulated (83 genes up vs. 13 genes down), clearly suggesting that DSM 16656^T^ responded by increased motility to root exudate exposure (Supplementary Table S3). Most importantly, we found the expression of genes of KFS1 but not of KFS2 to be differentially expressed (Figure 3B). Although being separated by more than a thousand genes, expression of both clusters of KFS1 was up-regulated (KFS1.a up to 22.3-fold, KFS1.b up to 29.4-fold). In contrast, expression of genes encoding the master regulator of KFS2 was significantly down-regulated (*flhC* 23.9-fold and *flhD* 26.8-fold). Hence, we concluded that KFS1 but not KFS2 is required for bacterial swimming toward the host. The active down-regulation of the KFS2 master regulator suggests that KFS2 biosynthesis has to be avoided in the tested environment.

### *K. radicincitans* DSM 16656^T^ alters the bacterial community composition of plants

**Figure 6:**
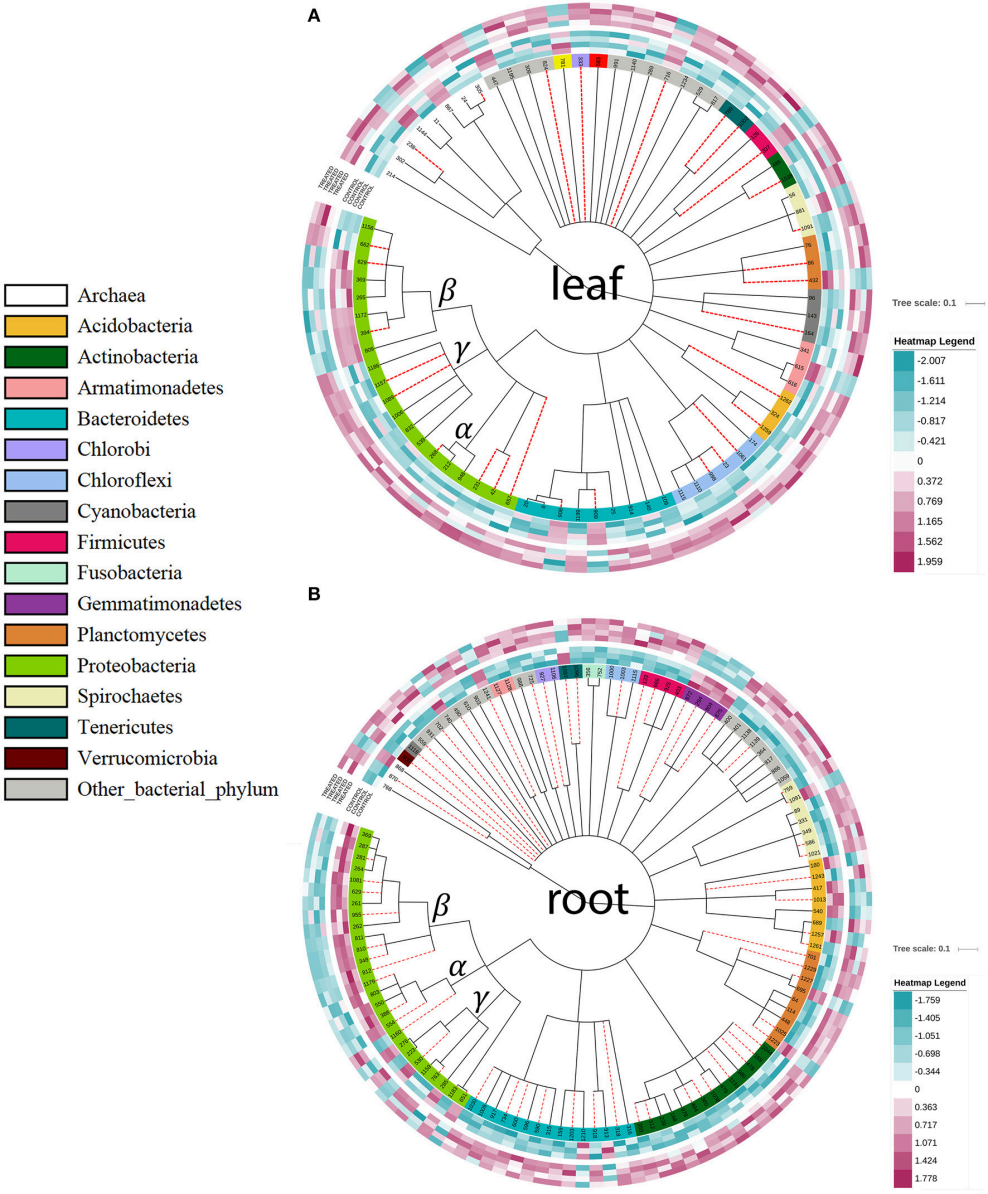
PhyloChip analysis of the bacterial community composition of tomato plants 14 dpi with *K. radicincitans* DSM 16656^T^. Operational taxonomic units (OTUs) with significantly changed abundance values; outermost circles represent the four replicates of tomato plants treated with *K. radicincitans*; inner circles represent the controls; purple colored boxes represent increased OTU abundance and blue colored boxes decreased OTU abundance. **(A)** leaf samples; **(B)** root samples. Red dashed lines, unclassified taxa; α, β, and γ, subgroups of Proteobacteria.

To test our second hypothesis that the three distinct type VI secretion systems (T6SS.1-3) enable *K. radicincitans* to exert significant influence on its environment, e.g., by suppressing other bacteria competing for the same ecological niche, we performed a PhyloChip analysis. Two weeks after inoculating *K. radicincitans* DSM 16656^T^ into leaves and roots of young tomato plants the bacterial and archaeal community composition was significantly affected (Figure 6). The presence of DSM 16656^T^ was confirmed by qPCR; four days after inoculation the average concentration of DSM 16656^T^ was 2.44E+07 cells per g fresh weight of leaf tissue and 2.88E+06 cells per g fresh weight of root tissue (Supplementary Figure S8). In total 1,220 operational taxonomic units (OTUs) could be detected, of which 78 (belonging to 33 families) showed significantly altered abundance in leaves (Figure 6A), and 122 (belonging to 76 families) showed significantly altered abundance in roots (Figure 6B). Performing Welch's *t*-test the number of significantly affected OTUs increased to 153 in leaves (13%) and 194 in roots (16%). Most of these OTUs displayed increased abundance in both leaf and root samples when inoculated with *K. radicincitans*, but the Betaproteobacteria clearly differed from the overall picture showing decreased abundance in leaves and roots. Interestingly, Alphaproteobacteria showed a decreased abundance in roots, but increased abundance in leaves.

### *K. radicincitans* DSM 16656^T^ employs the same colonization pattern in different plant species

**Figure 7:**
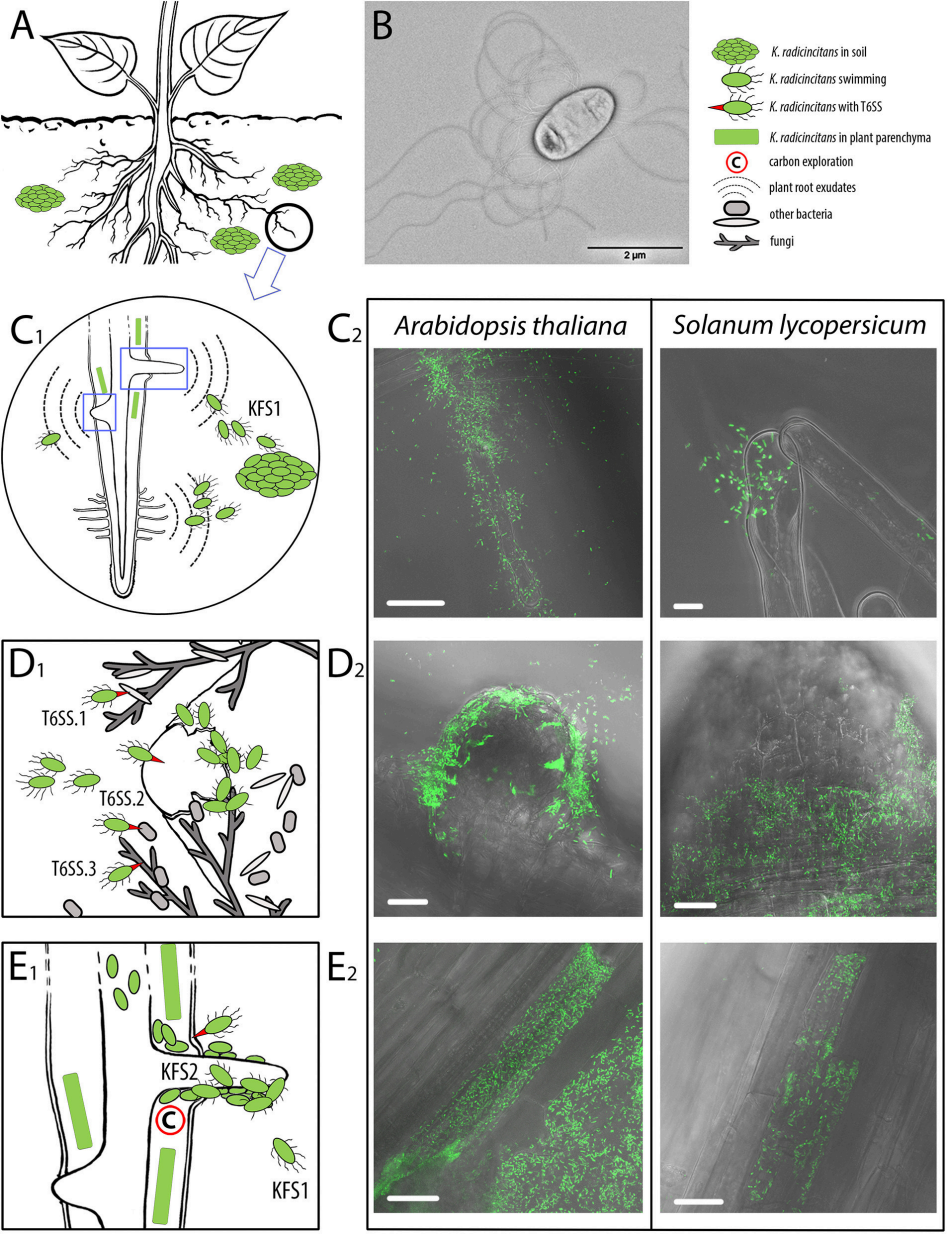
Plant root colonization scenario of *Kosakonia radicincitans* and supporting micrographs. **(A)** Supposed survival of *Kosakonia radicincitans* aggregates in the soil. **(B)** Transmission electron micrograph of peritrichously flagellated cell of *K. radicincitans* DSM 16656^T^ cultivated on semi-solid agar for 24 h. **(C1)**, **(D1)**, and **(E1)**. Schematic drawings illustrating the colonization steps of *K. radicincitans*. **(C2)**, **(D2)**, and **(E2)**. Confocal laser scanning micrographs of (eGFP) expressing DSM 16656^T^ colonizing roots of *Arabidopsis thaliana* and *Solanum lycopersicum* 6 dpi. **(C)** Plant roots exudate organic compounds that attract *K. radicincitans* toward root hairs and lateral roots. *K. radicincitans* has two flagellar systems (KFS1 and KFS2); when exposed to root exudates KFS1 of *K. radicincitans* enables high swimming motility and possibly attachment to plant surface. **(D)** *K. radicincitans* is particularly attracted by newly emerging lateral roots and colonizes intercellular spaces of root parenchyma via cracks around lateral roots; three distinct type VI secretion systems (T6SSs) help to interact with both plant host (evasion of plant immunity) and associated microbiota. **(E)** Migration into plant parenchyma cells supported by T6SSs injecting effector proteins. The scenario of *K. radicincitans* cells switching from KFS1 to KFS2 when attaching to plant surface and colonizing the roots is speculative; the purpose of KFS2 has not been determined yet. Note that proportions of plant, fungi and bacteria are not to scale. Green rectangle, root parenchyma cell densely colonized by *K. radicincitans*. White scale bar 20 μm.

To determine the motility and plant colonization potential of DSM 16656^T^ we performed microscopical analyses (Figure 7). Visualizing flagella of bacterial cells by transmission electron microscopy (TEM), *K. radicincitans* DSM 16656^T^ was found to be peritrichously flagellated with flagella approximately 10 μm long (Figure 7B). For elucidating the places of plant colonization, we generated green fluorescent protein (eGFP) expressing mutants of DSM 16656^T^ and studied zones of bacterial agglomeration on and inside roots of tomato (asterids group) and *Arabidopsis thaliana* (rosids group). DSM 16656^T^ was able to colonize both plant species in the same manner: being initially attracted by root hairs (Figure 7C), it entered the plant by cracks surrounding newly emerging lateral roots (Figure 7D), and eventually colonized the interior of individual root parenchyma cells (Figure 7E, Supplementary Videos 1, 2). Although complete colonization of plant parenchyma cells was infrequently observed, it could nonetheless be documented in both the distantly related plant species. Strong expression of eGFP indicated that the bacteria were still metabolically active, i.e., not destructively affected by plant defense.

## Discussion

### Phylogenetic and comparative genome analyses of *Kosakonia*

Our phylogenetic analyses confirm results of other working groups that there are two main clades of *Kosakonia* (Brady et al., 2013; Gu et al., 2014). Our comparative genome and functional classification analyses exhibit clade-specific features beyond 16S rRNA gene sequences and “housekeeping” genes. Two outstanding genomic features of the KORA (= Kosakonia radicincitans) clade conserved among strains of this species but missing in most other PGPB are an additional flagellar system (KFS2) and an additional type VI secretion system (T6SS.3). We considered the consistency of these two gene clusters in *K. radicincitans* to reflect important functions and asked the three following questions: (1) Why has *K. radicincitans two* flagellar systems? (2) Does *K. radicincitans* affect the natural microbiota of plants (possibly via T6SSs)? (3) How and where is *K. radicincitans* colonizing the plant?

### Genomic features of PGPB adaptation to plants

The PGP capability of bacteria is supposed to be conferred by genes involved in e.g., nitrogen fixation, mineral nutrient solubilization or phytohormone production. Although some of the underlying gene clusters have been studied in detail, the purpose of others, such as the *anf* operon, is still puzzling (Yang et al., 2014). On the other hand can some capabilities of bacteria not be explained by the presence or absence of genes *per se*. The ability to solubilize rock-phosphate or calcium-phosphate has been attributed to the *pqq* operon, which is missing in DSM 16656^T^, with the exception of a *pqqE*-like gene on the large plasmid (Figure 4B). While the missing *pqq* operon would suggest a limited ability of this strain to solubilize rock phosphates, recent experiments have proven that DSM 16656^T^ is capable of solubilizing an entire range of phosphates such as Ca_3_(PO_4_)_2_, Fe_3_(PO_4_)_2_, AlPO_4_ and even hydroxyapatite (Supplementary Figure S9). It will be interesting to see whether other genes have adopted the role of missing *pqq* genes in DSM 16656^T^ and whether they form a functional unit together with *pqqE* from the large plasmid.

In order to tap their PGP potential bacteria need to be in contact with the plant. For plant-associated bacteria the rapid colonization of the plant is crucial for establishing a metabolically active population, i.e., a population of sufficient “quorum sensing density” (Hartmann et al., 2014). The requirement of bacterial chemotactic motility for establishing a bacterial-plant association is well-known and was shown long ago (Vande Broek et al., 1998). Many bacterial strains of the family *Enterobacteriaceae* were reported to be (i) rapidly moving (Wolf et al., 2015) and (ii) plant growth-promoting (Lin et al., 2012), tempting us to speculate that soil-borne enteric PGPB may rely on exceptional motility in order to approach and colonize plants before other bacteria do. Colonization of the host is preceded by swimming toward host attractants (root exudates) using powerful flagella. Arriving at the plant surface some bacteria use pili to promote attachment to specific host cell receptors and apply secretion systems to inject effector proteins into the host in order to evade host immunity. Once the niche at the host plant has been reached the colonizing bacteria must compete with other microorganisms for this rare habitat. Possessing three T6SSs, we suspect *K. radicincitans* has an appropriate answer to many different environmental cues. Two general observations on the distribution of type VI secretion systems (T6SSs) suggest that the species-wide occurrence of three T6SSs in *K. radicincitans* is extraordinary: (i) T6SSs occur in only one quarter of bacteria (Bingle et al., 2008) and (ii) only 7% of T6SS-containing bacteria possess three or more T6SSs (Bernal et al., 2018). T6SSs have long been considered virulence factors of pathogenic bacteria, but next to evasion of host immunity they have meanwhile been associated with interbacterial competition for niches and survival in harmful environments (Wang et al., 2015).

“The wide distribution of T6SS clusters in plant-associated bacteria suggests that this molecular weapon is important for optimal fitness during plant colonization be it to cope with the plant response as much as with the resident microbiota” (Bernal et al., 2018). Based on genetic architecture and phylogeny T6SSs have been divided into five groups (Boyer et al., 2009), and searching for correlations between groups of T6SS and similar ecological niches Boyer and co-workers referred to group 4 as the group of “plant associated bacteria.” However, Bernal and co-workers found it more reasonable that the role of a specific T6SS is not defined by the group affiliation but primarily driven by the function of the effectors that the system secretes. Many effector-encoding genes are found in close proximity to *vgrG, hcp*, or *paar* genes and effectors are often fused to components of the Hcp–VgrG–PAAR structure; and VgrG, Hcp and PAAR-domain containing proteins (e.g., Rhs) may represent toxic compounds themselves (Cianfanelli et al., 2016). T6SS.3 of *K. radicincitans* possesses *vgrG, hcp* and *paar* genes (Supplementary Figure S7) indicating its large potential to eject effectors. Additional work will be required for detecting cargo effectors that are not fused with any component of the secretion machinery and, in some cases, are not genetically linked with any other T6SS genes (Cianfanelli et al., 2016). “The presence of non-T6SS-related elements encoded within or near T6SS clusters is an important factor to take into account” (Bernal et al., 2018). Interestingly, the genetic neighborhood of *K. radicincitans'* T6SS.3 harbors genes for multiple antibiotic resistance (*marR*), multidrug resistance (*mdtN, emrB*), and oxidative stress mitigation (*marR, katG*) suggesting that T6SS.3 provides fitness advantages in both polymicrobial and plant environments. The order of T6SS.3 genes in the KORA group is highly conserved (Figure 3) suggesting that T6SS.3 is of rather ancient origin in this species and may confer an important function to *K. radicincitans*.

Using the plant growth-promoting endophytic strain *Enterobacter cloacae* ENHKU01, Liu and co-workers performed antagonistic assays and found significant effects against pathogenic microbes within the environmental niche of ENHKU01, such as *Ralstonia solanacearum* and a wide range of fungal species (Liu et al., 2013). The authors hypothesized that *E. cloacae* strains possessing more than one distinct T6SS, such as ENHKU01, are likely to have fitness advantages in a broader range of habitats. Considering (i) that *K. radicincitans* possesses both T6SSs of ENHKU01 and another distinct T6SS (Supplementary Figure S7) and (ii) that T6SS likely provides fitness and colonization advantages to phytobacteria *in planta* (Bernal et al., 2018) we speculate that *K. radicincitans* benefits from its three T6SSs while colonizing its host.

### Multiple flagellar systems and multiple secretion systems may help to avoid pattern triggered immunity of plants

Microorganisms are known to trigger plant immunity via microbe-associated molecular patterns (MAMPs). Pattern-recognition receptors (PRRs) of plants recognize MAMPs and induce pattern-triggered immunity (PTI) that minimizes microbial invasion. In order to evade plant immunity microorganism respond in different ways. Given a mutualistic symbiosis, endophyte and host may co-evolve toward perception and signaling without triggering each other's immunity. This may also involve masking or non-synthesis of MAMPs such as chitin (Becker et al., 2016). In contrast, any plant-microbe interaction not based on long-term co-evolution is likely followed by defense upon microbe perception, initially yielding slower plant growth. However, *K. radicincitans* DSM 16656^T^ promotes growth of a wide range of plant species shortly (two weeks) after inoculation, suggesting an ability to circumvent or rapidly overcome perception by the plant and to avoid MAMP triggered immunity.

Investigating *Kosakonia radicincitans* strain YD4, Bergottini (2015) was the first to describe two “flagellar biosynthesis operons” in this species, but the dimension and rareness of the two flagellar systems among enteric bacteria was not determined. We found that all strains of *K. radicincitans* but only few strains of other enteric bacteria have two flagellar systems, here called KFS1 and KFS2 (with “K” referring to *Kosakonia*). KFS1 and KFS2 differ in gene cluster composition and in the amino acid sequence of their flagellin protein. The C-terminal end of the conserved Flg15 motif of the flagellin molecule is of high importance for perception by the plant. While synthetic peptides that lack the two amino acid residues present at the C terminus proved completely inactive as elicitors in *A. thaliana* (Bauer et al., 2001), in tomato abrupt loss of elicitor activity occurred for peptides lacking four amino acid residues (Felix et al., 1999). The substitution of two of the four last amino acid residues, which occurs in *E. coli* and *K. radicincitans* (Supplementary Figure S6), was shown to reduce the elicitor activity of Flg15 to < 1/3 when compared to Flg15 of *Pseudomonas aeruginosa* (Meindl et al., 2000). The substitution of the very last amino acid of Flg15 in KFS2 of *K. radicincitans* may further reduce its elicitor activity. A putative switch from KFS1 expression to KFS2 expression in the vicinity of plants would help *K. radicincitans* to avoid PTI of plants. However, expression of genes encoding pathogenesis-related (PR) proteins was induced in *Arabidopsis thaliana* treated with DSM 16656^T^ (Brock et al., 2013) suggesting that DSM 16656^T^ was recognized by the plant. Thus, we speculate, that effector proteins ejected by the three T6SSs of *K. radicincitans* are involved in interbacterial competition and plant immunity suppression likewise.

### *K. radicincitans* inoculation affects the microbiota of plants

Assuming a correlation between secretion systems, motility and low host specificity one may expect *Kosakonia* to strongly compete with the natural microbiota of plants for available niches. *K. radicincitans* DSM 16656^T^ was shown to possess high competitiveness and plant colonizing ability when applied to *Triticum aestivum*, its native host (Ruppel et al., 1992). Six weeks after inoculation wheat roots and shoots were equally colonized by about 10^6^ cells per mL root or shoot sap, irrespective of the inoculum density, i.e., 10^2^, 10^4^, 10^6^, or 10^8^ cells per plant. Even the non-host plant *Brassica oleracea* was densely colonized with *K*. *radicincitans* accounting for 10–16% of the total plant bacterial community 14 days after inoculation (Ruppel et al., 2006). Our PhyloChip analysis (Figure 6) showed that DSM 16656^T^ has a massive impact on the bacterial community composition of tomato. Whether *Kosakonia*'s competitor suppression is simply caused by niche exclusion via rapid plant colonization or by a more sophisticated approach including antibiotic compounds needs to be determined. However, type VI secretion systems are known for directly targeting eukaryotic cells, and also for interbacterial interactions in polymicrobial environments (Records, 2011; Ho et al., 2014; Alteri and Mobley, 2016; Cianfanelli et al., 2016). Microbiota analyses on a range of plants (other than tomato) will be required to see whether an inoculation of *K. radicincitans* shifts plant bacterial communities always in the same direction, irrespectively of the plant genotype, and whether fungi are also affected. Documenting the altered microbiota of plants during a time series will help to determine whether differences in the plant microbiota are compensated over time, or can be carried via seeds to the next plant generation, thus having a massive impact on the holobiont.

### Dual flagellar systems may allow increased motility in different environments

Bacteria are supposed to benefit from multiple flagellar systems due to increased motility in different environments (McCarter, 2004, 2005; Bubendorfer et al., 2014). Confronted by an antagonistic microorganism high motility may e.g., help to escape from destructive competition. However, in general dual flagellar systems were reported to be required for distinct modes of migration in different abiotic environments. For instance it was shown that some bacteria have primary and secondary flagellar systems for swimming and swarming (McCarter, 2004, 2005). According to the repertoire of motility genes, *Kosakonia* having two entire flagellar systems, *Enterobacter* having one and *Klebsiella* having only a fraction of one (Figure 2C), the three genera may differ in their potential to swim and swarm. Our studies have shown that the first flagellar system of DSM 16656^T^ (KFS1.a + KFS1.b) is expressed in liquid culture and upregulated in response to root exudates (Figure 3) indicating that KFS1 is used for swimming toward host plants; but the purpose of KFS2 has to be determined yet. *Kosakonia oryzae* Ola 51^T^ is closely related to *K. radicincitans* DSM 16656^T^ (Figure 1), possesses both flagellar systems (Figure 4) and like DSM 16656^T^ was found to be peritrichously flagellated (Li et al., 2017), but whether both flagellar systems contribute to formation of peritrichously inserted flagella still has to be investigated.

In addition to earlier reports, recent analyses have indicated that duplicated flagellar systems may be involved in biotic interaction. Contributing to competition for nodulation the secondary flagellar systems of *Bradyrhizobium diazoefficiens* (Alphaproteobacteria) is involved in plant colonization (Mongiardini et al., 2017). Levy and co-workers found that additional flagellum-like gene clusters are distributed within *Burkholderiales* (Betaproteobacteria) among plant- and soil-associated strains and concluded that this flagellar structure variant evolved in the plant environment (Levy et al., 2018). Moreover, the authors discovered that genomes of plant-associated bacteria encode more carbohydrate metabolism functions than related non-plant-associated genomes do. Interestingly, there are only five genes inserted into KFS2 of DSM 16656^T^ that do not directly belong to flagellar biosynthesis, functioning, and chemotaxis, and four of them encode proteins involved in carbohydrate metabolism. Among these four genes is *axe1-6A* that encodes a highly efficient carbohydrate esterase for degrading matrix polysaccharides (hemicellulose) in plant cell walls (Kabel et al., 2011). Our finding that there is a duplicated *mdtABC* operon (*mdtABC.2*) adjacent to KFS2, which is restricted to strains that possess KFS2, suggests a functional link of both gene clusters. Next to export of the siderophore enterobactin (Horiyama and Nishino, 2014) the MdtABC efflux pump was found to be involved in resistance to plant antimicrobials, such as flavonoids and tannin, and MdtABC-deficient mutants of *Erwinia amylovora* showed reduced ability to multiply in apple rootstock (Pletzer and Weingart, 2014). Although *Erwinia amylovora* is a plant pathogen, it is obvious that beneficial plant-associated bacteria would equally benefit from resistance to antimicrobial plant compounds. Assuming that *mdtABC.2* and *axe1-6A* of DSM 16656^T^ are involved in plant interaction it is tempting to speculate that KFS2 contributes to the plant-associated lifestyle of *K. radicincitans*.

### DSM 16656^T^ exhibits an endophytic, non-damaging lifestyle

Genes involved in degradation of the major cell wall components lignin and cellulose, next to chitin the most common organic compounds on earth, appear to be missing from *K. radicincitans*. Lignin is an aromatic polymer and as such a potential carbon source for enteric bacteria such as *Klebsiella* (Diaz et al., 2001), a genus that carries up to 10 times as many genes for metabolism of aromatic compounds as *Kosakonia* (Supplementary Figure S5). Nonetheless, the absence of lignin and cellulose degrading enzymes in *K. radicincitans* may refer to its endophytic, non-damaging lifestyle, which we infer from our observation that *K. radicincitans* has never caused any detrimental effect on host plants despite massive colonization. Interestingly, DSM 16656^T^ carries two chromosomal regions that contain several genes for cellulose synthesis (*bcsABCEZ, acsABCD, yhjDEHUT*), possibly important for biofilm formation and plant colonization (Römling and Galperin, 2015). These gene clusters are shared with a few other enteric bacteria, among them *Enterobacter* sp. FY-07 which was confirmed to produce bacterial cellulose (Ji et al., 2016).

### There is a strong link between *Citrobacter, Kosakonia* and other enteric PGPB

Genes shared between DSM 16656^T^ and *Citrobacter*, presumably resulting from horizontal gene transfer, have increased the adaptive potential of DSM 16656^T^. There is an integrative conjugative element (ICE) on the chromosome comprising 109 genes which is shared between DSM 16656^T^ and *Citrobacter amalonaticus* Y19, but missing for the most part in the other 33 bacterial genomes we analyzed (Figure 4A). The ICE contains a gene cluster (*pilLM/bfpB/pilO/epsE/tcpE/pilS*) for type IV pili formation, bacterial appendages that are involved in adhesion to host cells and biofilm formation. These pili or fimbriae are filamentous structures like flagella, but smaller, and are thought to enable gliding or twitching motility, a synchronized and flagella-independent form of bacterial movement over moist surfaces, important in rapid host colonization (Mattick, 2002).

The observation that most genes of the large plasmid of DSM 16656^T^ are shared with *Citrobacter* and missing in other *Kosakonia* strains is further evidence for the exchange of genomic material between both genera. The large genome-wide homology of *Enterobacter* sp. 638, an endophyte of poplar from USA, to *Citrobacter koseri* ATCC BAA-895 (Taghavi et al., 2010) suggest regular horizontal gene transfer between *Citrobacter* and other groups of enteric bacteria. The same accounts to the fact that *Enterobacter* sp. 638 was nested within a *Citrobacter* clade in our comprehensive 16S rRNA gene tree (Supplementary Figure S2). Some *Citrobacter* strains were found to be plant endophytes with plant protecting capacities and PGP potential (Wang et al., 2006; Mousa et al., 2015). Hence, they do likely share habitats with endophytic bacteria of genus *Kosakonia* and *Enterobacter* and may synergistically interact while exhibiting a beneficial impact on plants.

### The large plasmid may enhance the plant-associated lifestyle of DSM 16656^T^

Some bacteria are able to produce 1,2-propanediol (1,2-PD) by fermenting rhamnose and fucose, common sugars in plant cell walls (Badia et al., 1985), and the genome composition of DSM 16656^T^ indicates that *K. radicincitans* is among these bacteria. However, 1,2-PD itself is a valuable carbon source and can be degraded by DSM 16656^T^. Intermediate products of 1,2-PD degradation are mutagens and need to be detoxified by coenzyme B_12_ in bacterial microcompartments (Sampson and Bobik, 2008). Consistently, genes for coenzyme B_12_ metabolism are provided by the large plasmid and the chromosome. Ongoing work has to clarify whether this anaerobic degradation of plant compartments is involved in establishing a microaerobic endophytic niche for N_2_-fixation or simply in decomposing dead plant material. 1,2-PD degradation is likely to provide a selective advantage in the anaerobic depths of soils (Sampson and Bobik, 2008), and “*Enterobacter lignolyticus*” SCF1, isolated from anaerobic tropical forest soils (DeAngelis et al., 2011), was the only *Enterobacter* strain found to share the bulk of *pdu* genes with DSM 16656^T^ (Figure 5). However, intermediate and final products of PDD and GT may also be involved in better plant performance, both the volatile organic compounds 1,2-propanediol and 1,3-propanediol were reported to promote plant growth (Nakajima et al., 2005; Tahir et al., 2017). Taken together, the genomic composition of *K. radicincitans* underpins its tight interaction with plants, but the large plasmid of DSM 16656^T^ appears to have further enhanced the plant-associated lifestyle of this particular strain.

### Multiple rRNA operons may allow responsiveness to diverse environments

Next to pili, flagella and secretion systems, another bacterial component has been reported to increase the fitness of bacteria: rRNA operons (*rrn*), of which DSM 16656^T^ possesses seven copies (Supplementary Figure S1B). The persistence of multiple *rrn* copies has been considered to reflect ecological strategies of bacteria. Klappenbach and co-workers discovered that soil bacteria grew rapidly on nutritionally complex medium and tolerated herbicide 2,4-dichlorophenoxyacetic acid (2,4-D) when *rrn* copy number was high (Klappenbach et al., 2000). The maximum reproductive rate of bacteria doubles with a doubling of *rrn* copy number, and the efficiency of carbon use is inversely related to maximal growth rate and *rrn* copy number (Roller et al., 2016). These reports suggest that bacteria with a high *rrn* copy number must exploit many nutrient sources but are rewarded by fast growth.

### Bacterial N_2_-fixation promotes plant growth, which in turn provides larger habitats to endophytic bacteria

The most striking difference between *Kosakonia, Klebsiella*, and *Enterobacter* in terms of PGP traits is the ability to fix atmospheric nitrogen: *Kosakonia* has two gene clusters for N_2_-fixation, *Klebsiella* has one and *Enterobacter* has none. Whereas, *nifHDK* of *K. radicincitans* encodes a component of the iron–molybdenum (FeMo) nitrogenase, which is found in all diazotrophs, its *anfHDGK* encodes for a component of the iron-only (Fe) nitrogenase found secondarily in only some diazotrophs (Ekandjo et al., 2017). In conformity with two nitrogenase gene clusters there are also two genetic components for regulatory cascades controlling *nif* transcription in *K. radicincitans*. Diazotrophic Gammaproteobacteria and rhizobia differ in the first level of the cascade, a two-component-system comprising the genes *ntrBC* and *fixJL*, respectively (Dixon and Kahn, 2004). While *Klebsiella* carries the *ntrBC* system and rhizobia the *fixJL* system, *Kosakonia* has both of them. Unlike *ntrBC, fixJL* is responsive to oxygen (Dixon and Kahn, 2004) guaranteeing that *nif* transcription only happens in a microaerobic habitat. Legume-rhizobia symbiosis is thought to start when particular plant root exudates bind to NodD, the transcriptional regulator of bacterial nodulation (Mus et al., 2016). Although not inducing nodule organogenesis, *K. radicincitans* DSM 16656^T^ carries two *nodD* genes and each of them is spatially close to either of the two-component systems controlling *nif* transcription. Hence, the presence of *fixJL* and *nod*D genes suggests that N_2_-fixation of DSM 16656^T^ is affected by plants and O_2_ concentration. Ekandjo and co-workers could not detect significant contribution of the Fe nitrogenase to biological dinitrogen assimilation under pure bacterial culture experimental conditions but speculated that having both nitrogenases might enable *K. radicincitans* to fix atmospheric nitrogen in different environments, however fixation of atmospheric nitrogen *in planta* was not studied (Ekandjo et al., 2017). Our microscopic analyses of DSM 16656^T^ colonizing root parenchyma cells of plants (Figure 7) suggest that enteric bacteria may be capable of generating their own microaerobic niche for atmospheric nitrogen fixation. A combination of microscopic and functional analyses is required in order to determine whether plant parenchyma cells are the location of *Kosakonia*'s N_2_-fixation.

### Costs and benefits of multiple gene clusters

Next to atmospheric nitrogen fixation other metabolically expensive gene clusters, such as dual flagellar systems, triple T6SSs (Figures 3, 4), and multiple rRNA operons (Supplementary Figure S1B) suggest that *K. radicincitans* DSM 16656^T^ encounters nutrient-rich environments, where high metabolic costs are not detrimental but outweighed by benefits. Addressing the need for high amounts of energy, *K. radicincitans* DSM 16656^T^ exhibits a genetic composition well-suited for exploiting a large variety of carbon sources. Pathways involved in oxidative phosphorylation are the only pathways shared between the chromosome and both plasmids of *K. radicincitans* DSM 16656^T^, probably indicating that generating sufficient energy is pivotal for this strain. Being a generalist plant colonizer, *K. radicincitans* must be highly adaptable to plant immunity systems in order to achieve host colonization. In this context, it will be interesting to see whether *Kosakonia* from the nutrient-poor soil of sundew (*Drosera burmannii*; Supplementary Table S1) also exhibits low host specificity and multiple copies of the gene clusters mentioned above.

## Summary and outlook

In this article we provide comprehensive knowledge about the newly emerging PGP species *Kosakonia radicincitans* and reveal duplicated species-specific gene clusters that have not yet been described in other bacteria. The competence of *K. radicincitans* to colonize and exert growth-promoting effects in a wide range of plants is intriguing and might be facilitated by genes contributing to high motility (two flagellar systems) and high competitiveness (three T6SSs). These genes now need to be functionally analyzed. Studies on the PTI capacities of *K. radicincitans* knock-out mutants, in which single T6SSs are deleted, will be required to answer the question, whether T6SSs found in *K. radicincitans* DSM 16656^T^ are involved in plant immunity suppression, in interbacterial competition, or both. Likewise, experiments on KFS knock-out mutants will be required to reveal the purpose of KFS2. More research on *K. radicincitans* strains other than DSM 16656^T^ is required in order to unravel the plant-colonizing and growth-promoting capacity of the whole species. Given that *K. radicincitans* exhibits unique, complex, and intra-specific consistent gene clusters this species has the potential to become a model organism for studies on multiple modes of motility and bacteria-host interactions.

Garcia-Fraile and co-workers stated that the use of “Enterobacteria” as biofertilizers involves a risk to humans and plants (Garcia-Fraile et al., 2012). However, the fact that natural hosts of *Kosakonia* belong to the most consumed crop plants in the world indicates that in general *Kosakonia*'s potential for causing plant and human diseases must be low. Although being closely related to pathogenic strains the potential of presumably beneficial bacteria has to be determined since the percentage of highly efficient PGPB among cultivable endophytic bacteria is low. We discovered a rate of 0.5% when we tested a thousand isolates from the surface-sterilized phyllosphere of winter wheat (Ruppel, 1988). This emphasizes the need for the synergistic use of bioinformatics for *in silico* prediction and affirmative *in vivo* experiments in order to distinguish promising candidates from really effective PGPB and also from possibly pathogenic strains. The comprehensive genome comparisons by Levy and co-workers found that plant-associated bacteria from across the bacterial kingdom share gene clusters for nodulation, nitrogen fixation, chemotaxis, flagellum biosynthesis and type VI secretion systems (Levy et al., 2018). Independently, we found the same gene clusters multiplied in *K. radicincitans* DSM 16656^T^, while searching for unique genomic features of an extraordinary successful colonizer and growth promoter of plants.

A database assigning PGP traits, e.g., a gene ontology (GO) term “plant growth-promotion,” would enable pre-selection of putatively beneficial microorganisms from a list of all isolated and fully sequenced ones. Deciphering the PGP potential of microorganisms by *in silico* analyses would significantly facilitate the future search for promising candidates for application in sustainable crop production.

## Author contributions

MB designed research and wrote the manuscript. SP and MB performed *in silico* analyses. YB generated eGFP mutants of DSM 16656^T^ and performed CLSM analyses. BeB performed *in vivo* experiments and generated material for microarray and PhyloChip analyses. MD processed microarray data. JO, BoB, and CS sequenced the genome of DSM 16656^T^ by SMRT and Illumina sequencing. JR performed TEM analyses, GT assessed phosphate solubilization activity. SR initiated microarray and PhyloChip analyses. MB, SP, YB, BeB, and SR analyzed the data. All authors contributed to writing the manuscript.

### Conflict of interest statement

The authors declare that the research was conducted in the absence of any commercial or financial relationships that could be construed as a potential conflict of interest.
