# Supplementary material for: Comparative Genomics Reveal a Flagellar System, a Type VI Secretion System and Plant Growth-Promoting Gene Clusters Unique to the Endophytic Bacterium Kosakonia radicincitans
Source: Front Microbiol. 2018 Aug 30;9:1997. doi: 10.3389/fmicb.2018.01997 (PMC6125372; doi:10.3389/fmicb.2018.01997)
Supplement: Supplementary file 3 [file Data_Sheet_1.pdf]

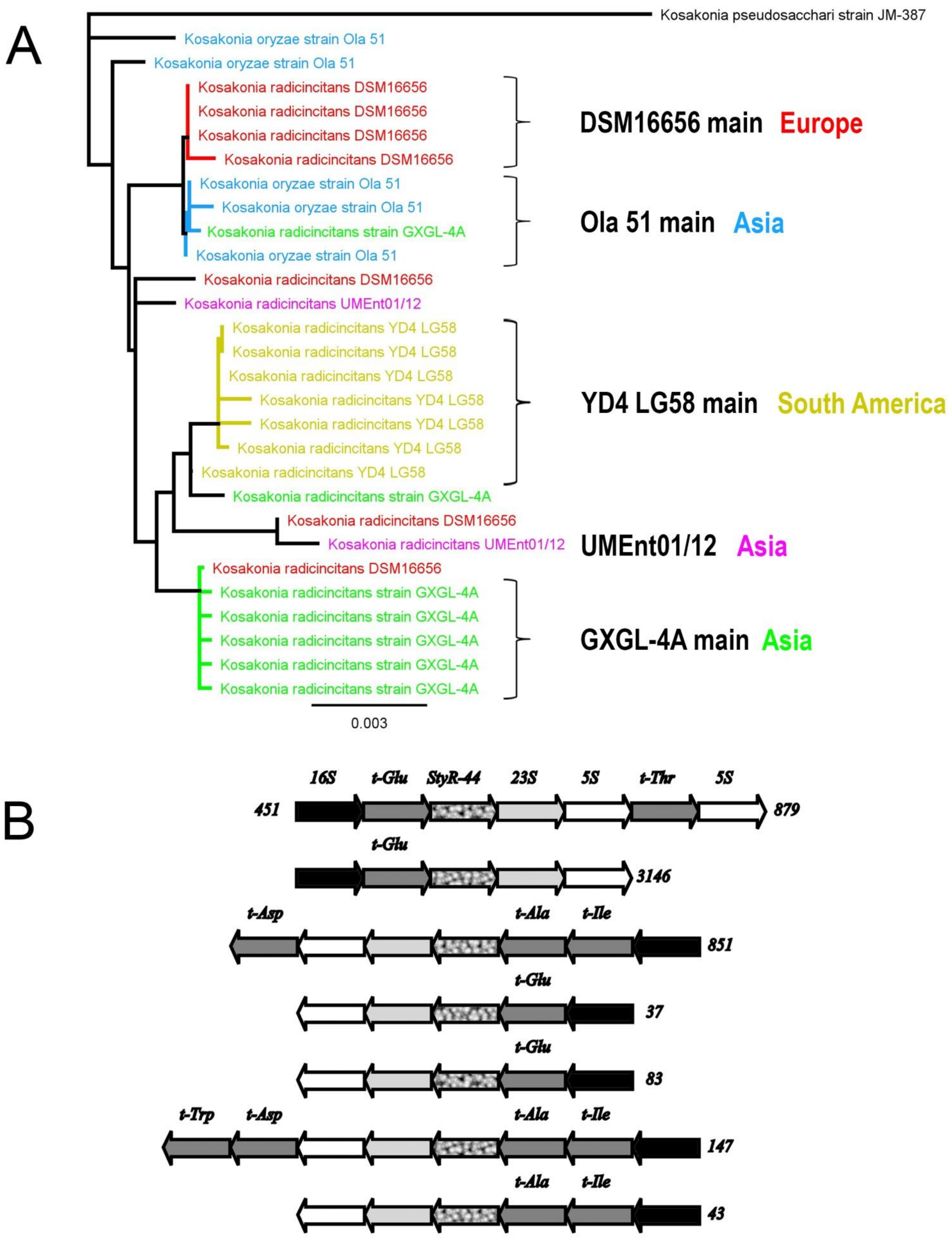

Fig. S1: Multiple copies of ribosomal RNA genes in *Kosakonia radicincitans*. **(A)** Phylogeny of multiple 16S rRNA gene copies of five *K. radicincitans* strains. **(B)** Seven rRNA operons of *Kosakonia radicincitans* DSM 16656^{T} differing predominantly in absence/presence of inserted tRNA genes.

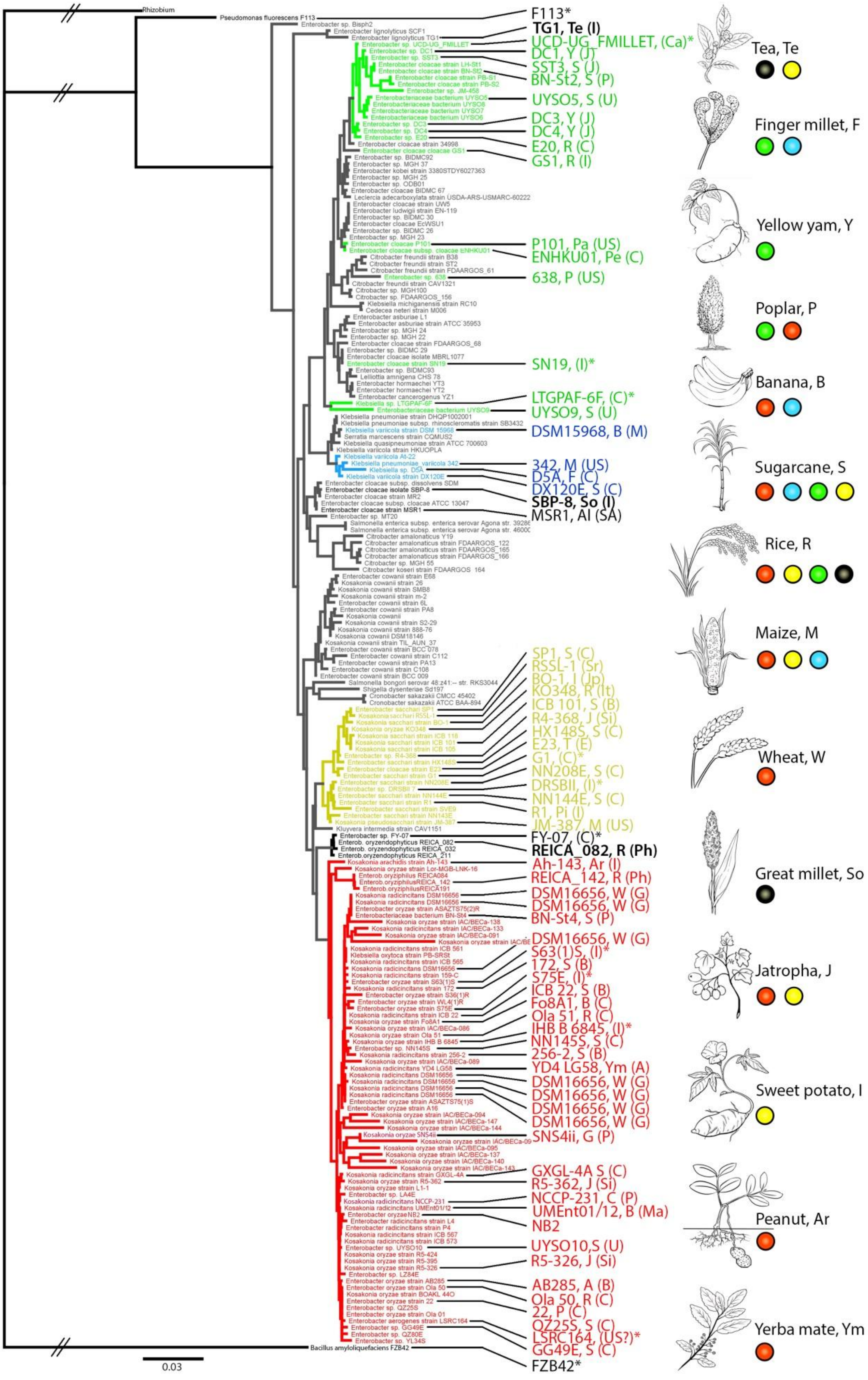

Fig. S2: 16S rRNA gene tree of most to date known *Kosakonia* strains and closely related PGP *Enterobacter* and PGP *Klebsiella* strains. Four major groups are considered and depicted in different colors in conformity with **Figure 1**: red = *Kosakonia radicincitans* (KORA) group, yellow = *Kosakonia sacchari* (KOSA) group, green = *Enterobacter* (ENTERO) group, blue = *Klebsiella variicola* (KLEVA) group. Host plants are marked by accordingly colored circles. Strains in bold black letters were found in plants marked by black circles. Capital letters after accessions refer to plant hosts, which are further characterized in **Table S1**. Capital letters in brackets refer to countries given in **Table S1**.

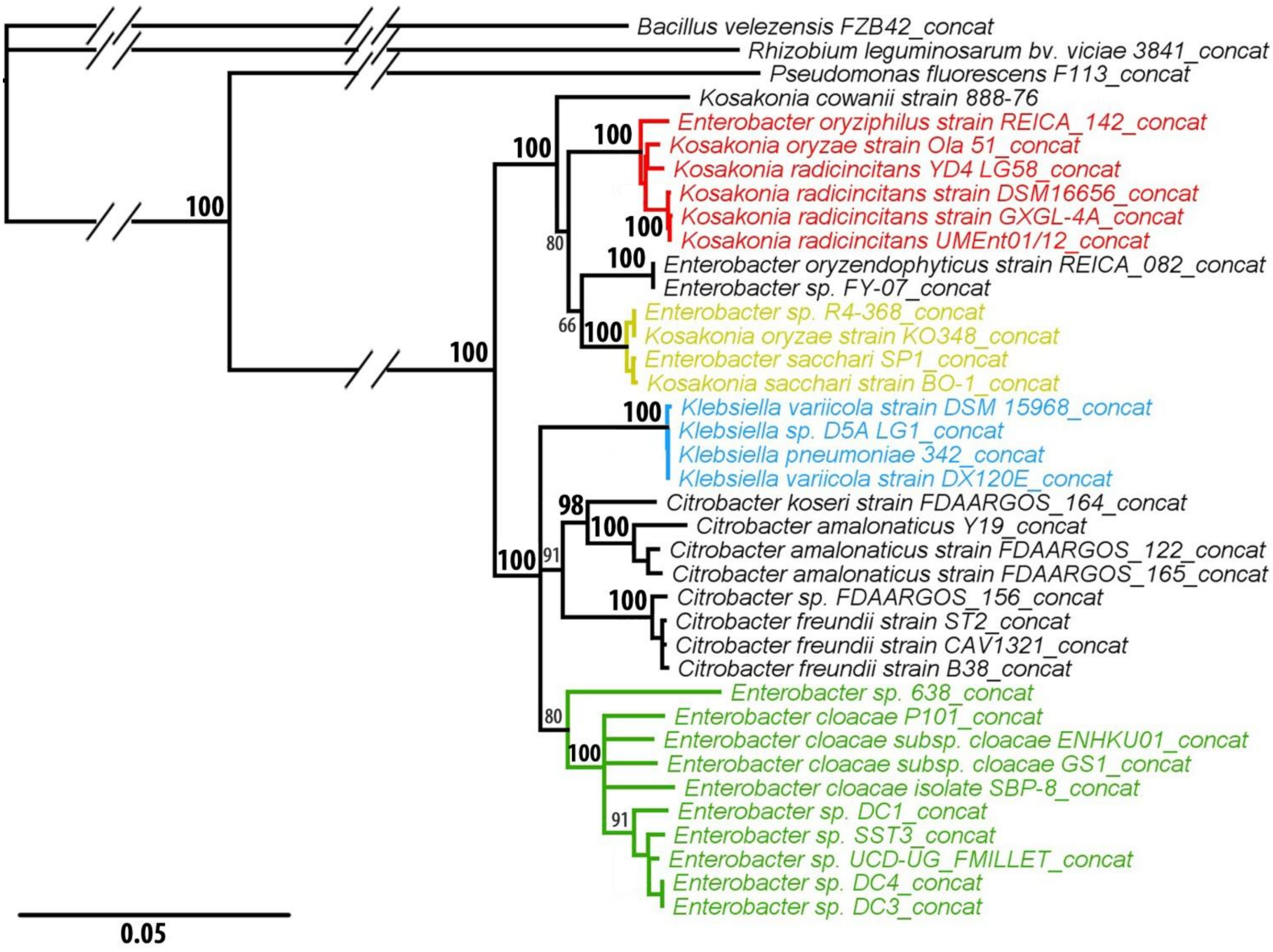

Fig. S3: Phylogeny based on concatenated amino acid sequences of housekeeping genes *atpD*, *gyrB*, *infB* and *recA*, considering only taxa with fully sequenced genomes. Bootstrap support is given for a selection of clades only.

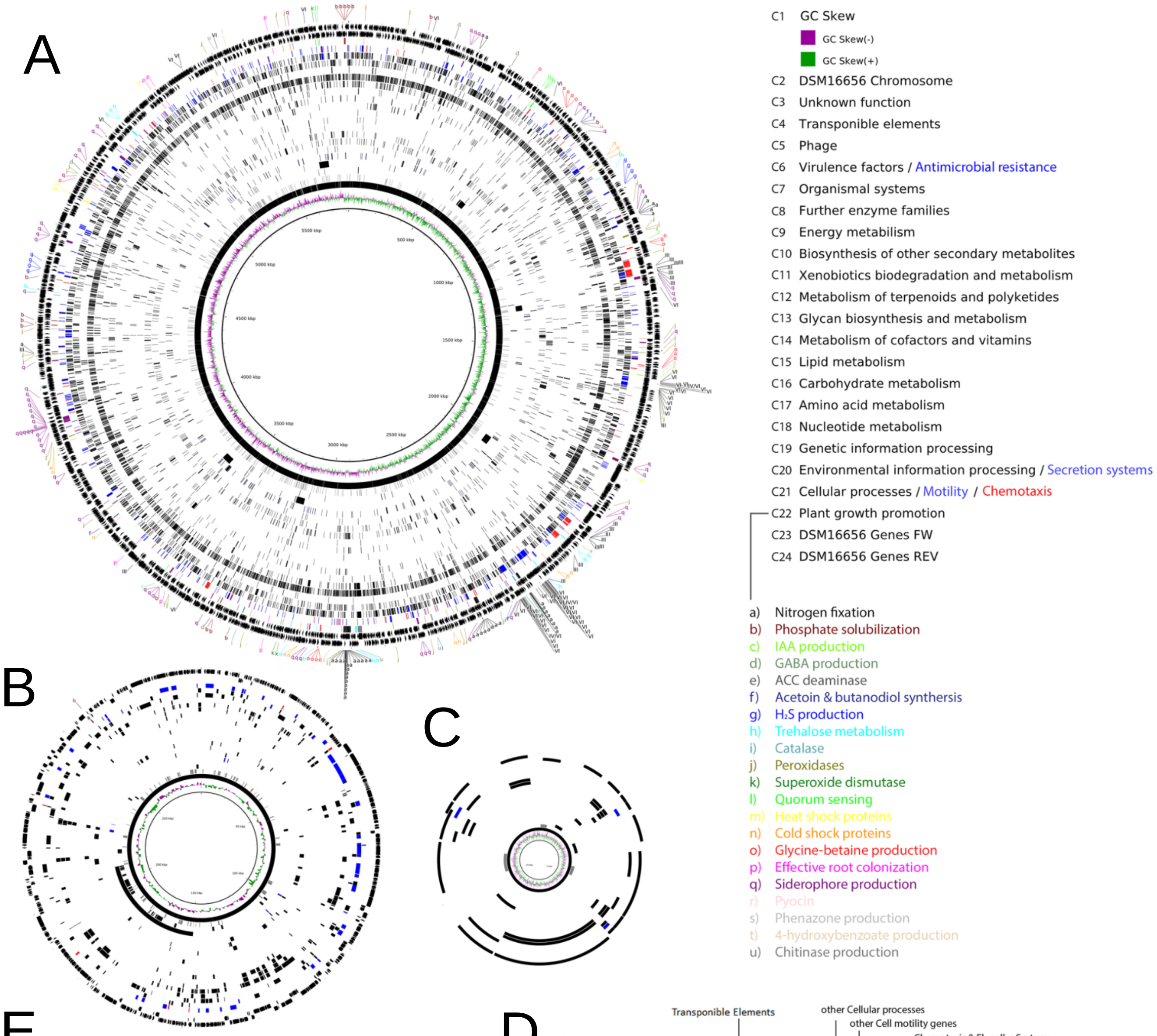

E

Functional category	number of genes	percentage of genome*
Cellular processes	518	8.6
>Cell motility	214	3.5
>>Chemotaxis & Flagellar system	166	2.7
Environmental information processing	1800	29.7
>Secretion systems	288	4.8
>>Type I secretion system	4	
>>Type II secretion system	11	0.2
>>Type III secretion system	20	0.3
>>Type IV secretion system	2	
>>Type IV / VI secretion system	3	
>>Type V secretion system	0	
>>Type VI secretion system	55	0.9
Virulence factors	232	3.8
>Antimicrobial resistance	120	2.0

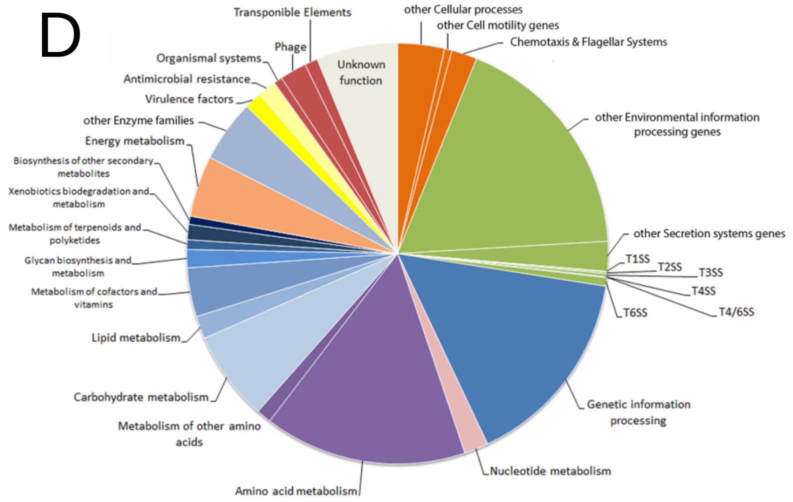

Fig. S4: Genome map of *Kosakonia radicincitans* DSM 16656^{T} and functional annotation. Circles C4 to C21 represent each a particular biological function; black lines within a circle represent genes. Colored genes depicted in circle C22 are involved in plant growth-promotion (see **Table 1**). The outermost circle of the chromosome map refers to PGP genes from circle C22 and provides information on secretion systems. **(A)** Chromosome, **(B)** Large plasmid, **(C)** Small plasmid. **(D)** and **(E)** Functional classification by BlastKOALA tool from KEGG of all annotated genes of *Kosakonia radicincitans* DSM 16656^{T}. **(D)** High-ranking functional categories and a selection of lower-ranking categories. **(E)** A selection of functional categories from (**D**); ***** percentages are given in relation to the total number of genes, not in relation to the number of coding sequences.

A

Category	KORA			KOSA			ENTERO			KLEVA			percentaged STDEV of all 12 taxa
	DSM16656	OLA 51	YD4	KO348	SP1	BO-1	P101	SST3	DC1	DSM15968	342	DX120E	
Amino Acids and Derivatives	547	483	526	419	438	439	510	483	479	586	600	593	12,0%
Carbohydrates	723	645	659	584	616	616	688	587	576	874	883	859	16,4%
Cell Division and Cell Cycle	40	36	38	36	36	36	41	38	38	37	42	46	7,8%
Cell Wall and Capsule	237	198	223	218	217	216	221	217	217	194	190	204	6,3%
Cofactors, Vitamins, Prosthetic Groups, Pigments	351	299	343	323	320	320	266	269	268	362	365	373	11,1%
DNA Metabolism	136	114	116	124	113	107	136	120	114	109	128	123	8,6%
Dormancy and Sporulation	4	4	4	4	4	4	4	4	4	5	5	5	10,1%
Fatty Acids, Lipids, and Isoprenoids	166	168	166	136	134	134	135	129	133	138	137	132	10,2%
Iron acquisition and metabolism	78	77	76	52	71	71	84	63	83	71	73	71	14,3%
Membrane Transport	224	197	208	152	162	160	233	202	198	208	204	246	17,0%
Metabolism of Aromatic Compounds	14	12	12	6	6	6	41	37	39	84	84	82	90,0%
Miscellaneous	50	47	47	49	49	49	51	47	46	95	95	96	34,4%
Motility and Chemotaxis	195	197	189	132	156	130	157	102	99	11	11	11	55,5%
Nitrogen Metabolism	59	56	59	53	53	53	46	48	39	54	63	54	14,4%
Nucleosides and Nucleotides	139	119	137	107	109	110	125	115	121	137	137	138	10,1%
Phages, Prophages, Transposable elements, Plasmids	92	50	33	55	33	39	53	29	42	44	29	55	34,8%
Phosphorus Metabolism	56	50	53	57	54	54	49	50	50	55	55	56	5,2%
Potassium metabolism	30	29	27	28	30	30	34	34	35	33	33	33	8,4%
Protein Metabolism	303	280	307	265	291	291	307	287	290	296	298	304	3,9%
RNA Metabolism	233	224	227	228	231	230	215	237	212	255	252	251	6,0%
Regulation and Cell signaling	160	149	148	140	140	136	167	152	155	173	173	173	8,9%
Respiration	178	132	150	163	159	160	154	151	151	175	177	183	8,8%
Secondary Metabolism	4	4	4	4	4	4	5	5	5	5	5	5	11,5%
Stress Response	223	204	197	189	188	190	198	181	179	183	191	186	5,9%
Sulfur Metabolism	81	65	77	63	65	63	70	66	65	83	90	91	14,0%
Virulence, Disease and Defense	150	110	118	107	111	111	126	106	118	136	147	150	13,0%

B

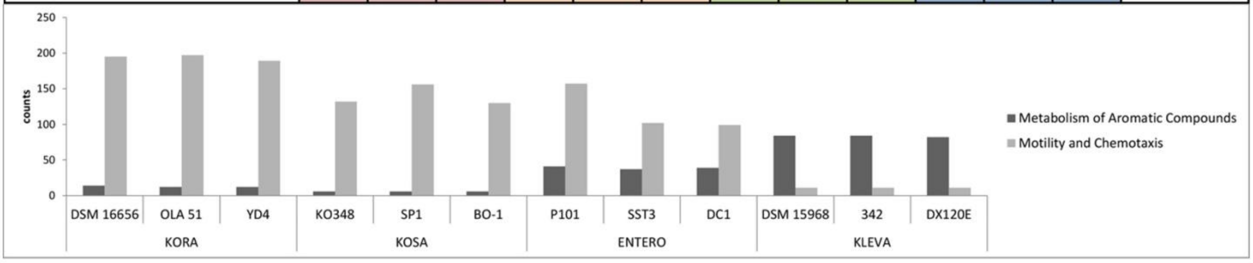

C

Motility and Chemotaxis	KORA			KOSA			ENTERO			KLEVA		
	DSM 16656	OLA 51	YD4	KO348	SP1	BO-1	P101	SST3	DC1	DSM 15968	342	DX120E
Flagellar Motility	37	39	37	27	32	26	31	21	20	0	0	0
Flagellum	93	94	93	54	72	55	84	47	45	0	0	0
Bacterial Chemotaxis	65	64	59	51	52	49	42	34	34	11	11	11
	**158**	**158**	**152**	**105**	**124**	**130**	**126**	**81**	**99**	**11**	**11**	**11**

D

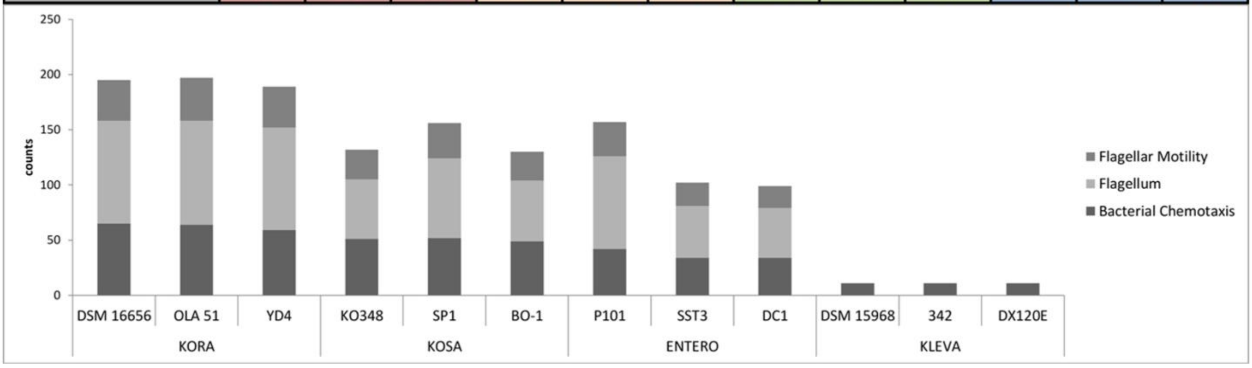

Fig. S5: Functional classification of DSM 16656^T, *Kosakonia radicincitans* (KORA group), *K. sacchari* (KOSA group), *Enterobacter* spp. (ENTERO group) and *Klebsiella variicola* (KLEVA group) by SEED using RAST-annotated genomes. **(A)** Number of genes from all categories assigned by SEED and depicted in **Figure 2**; last column shows the percentaged standard deviation in each category considering all 12 taxa: categories with percentaged standard deviation above 30% are highlighted; number of genes (= counts) of the two top categories with highest percentaged standard deviation ('metabolism of aromatic compounds' and 'motility and chemotaxis') are depicted in **(B)**. **(C)** Number of genes in sub-categories of 'motility and chemotaxis' category; **(D)** bar chart to **(C)**.

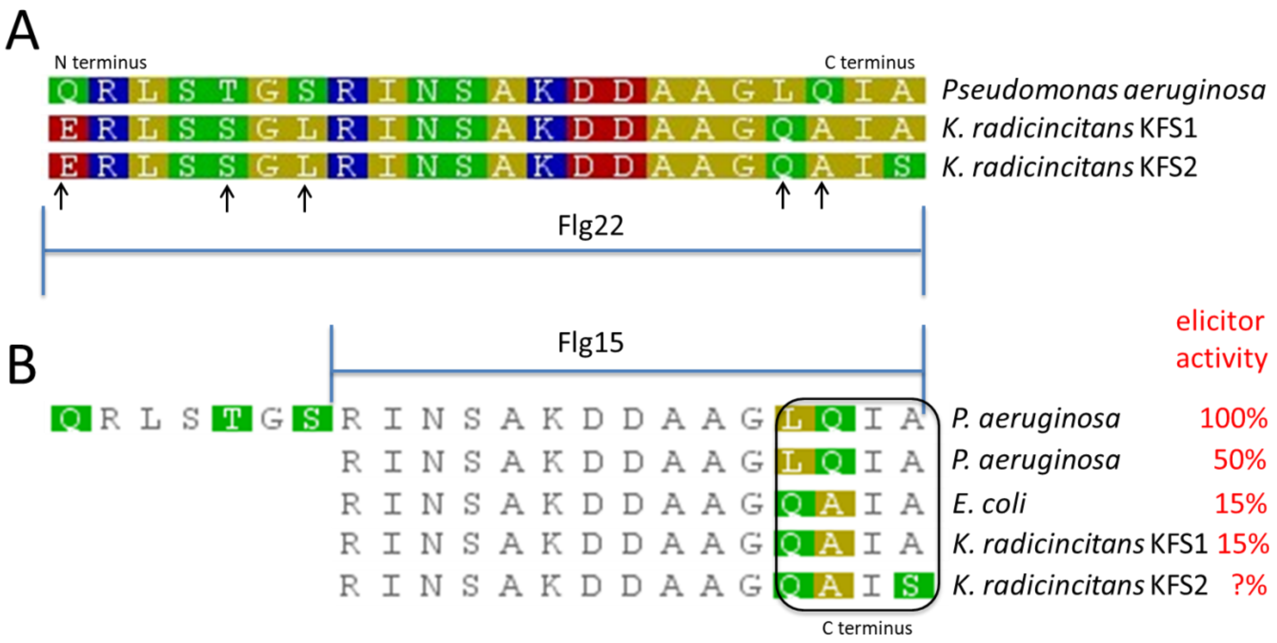

Fig. S6: Alignment of Flg22 and Flg15 peptide sequences of flagellins from both flagellar systems of *Kosakonia radicincitans* DSM 16656^{T} (encoded by *fliC* of KFS1 and *fliC* of KFS2) to Flg22 and Flg15 sequences of reference strains. **(A)** Polarity of Flg22 amino acids; arrows pointing to AA substitutions between *Pseudomonas aeruginosa* and *K. radicincitans*. **(B)** AA substitutions between *P. aeruginosa*, *E. coli* and *K. radicincitans* at C terminus of Flg15; percentages refer to alkalinization response of tomato cells to synthetic Flg22 and Flg15 peptides (= elicitor activity or PTI potential) according to Meindl et al. 2000.

Reference

Meindl T, Boller T & Felix G (2000) The Bacterial Elicitor Flagellin Activates Its Receptor in Tomato Cells According to the Address–Message Concept. *The Plant Cell* **12**: 1783-1794.

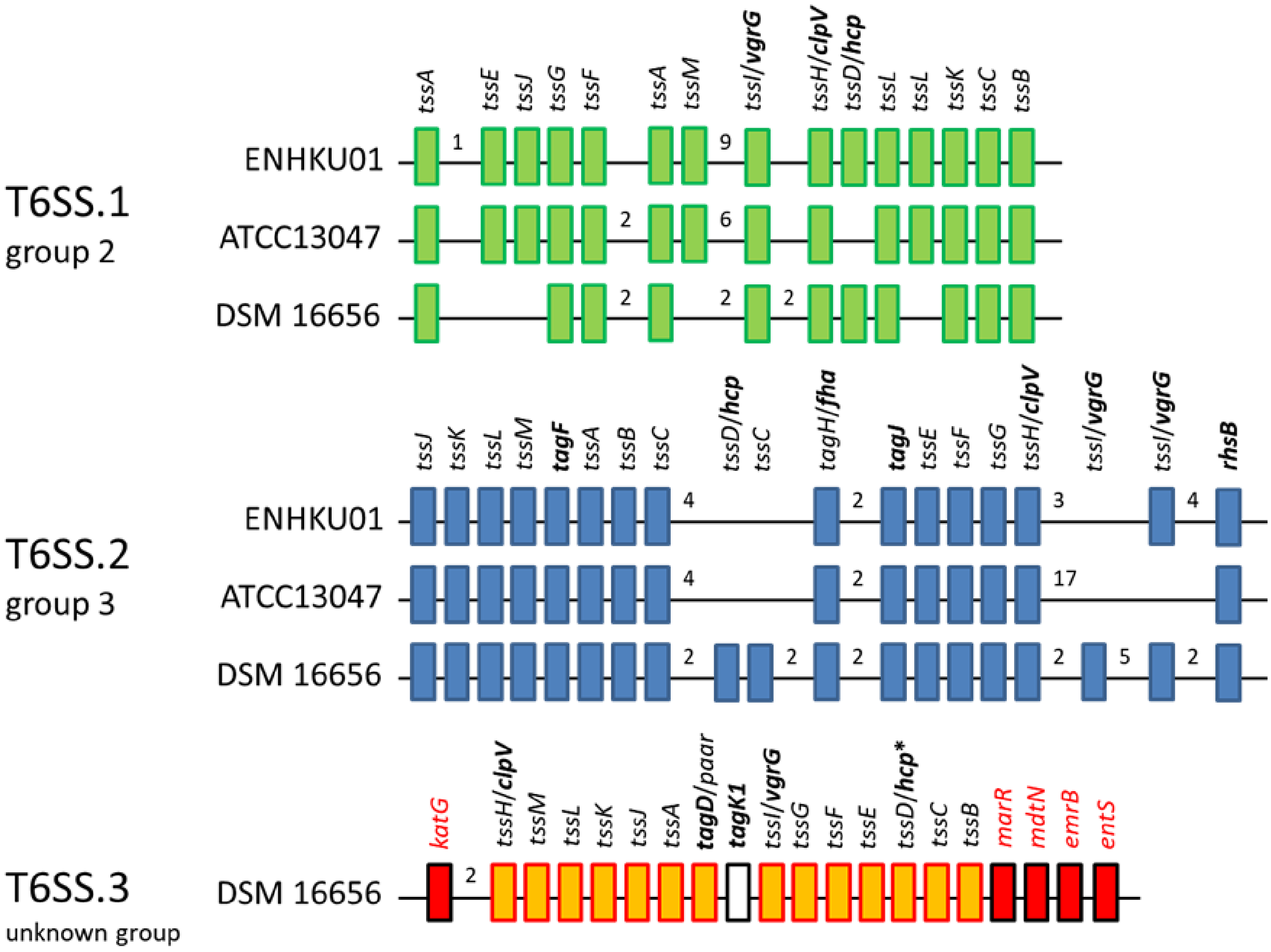

Fig. S7: Gene cluster composition of the three T6SSs of *Kosakonia radicincitans* DSM 16656[T] in comparison to two closely related *Enterobacter cloacae* strains. The *E. cloacae* strains exhibit the two T6SS clusters commonly found in *Enterobacter*; strain ENHKU01 is a plant growth-promoting endophyte and is included in the comparative genome analyses (**Figures 4 and 5**). T6SS composition of *Enterobacter* and the style of presentation was adapted from (Liu et al. 2013b), but simplified regarding variable regions; numbers refer to genes <u>not</u> belonging to *tss* or *tag* genes. Group 2 and group 3 refer to T6SS clusters found in plant-associated bacteria (reviewed in Bernal et al. (2018)); *paar* = gene encoding an Zn-binding Pro-Ala-Ala-Arg (PAAR) domain-containing protein; *tagK1* = *Kosakonia*-specific T6SS.3 accessory gene of unknown function; *hcp** = gene of Hcp1-like superfamily (IPR036624) identified by Interpro. Genes shown in red are from the genomic environment of T6SS.3 of *K. radicincitans* DSM 16656[T].

References

Bernal P, Llamas MA & Filloux A (2018) Type VI secretion systems in plant-associated bacteria. *Environ Microbiol* **20**: 1-15.

Liu WY, Wong CF, Chung KM, Jiang JW & Leung FC (2013) Comparative genome analysis of *Enterobacter cloacae*. *PLoS One* **8**: e74487.

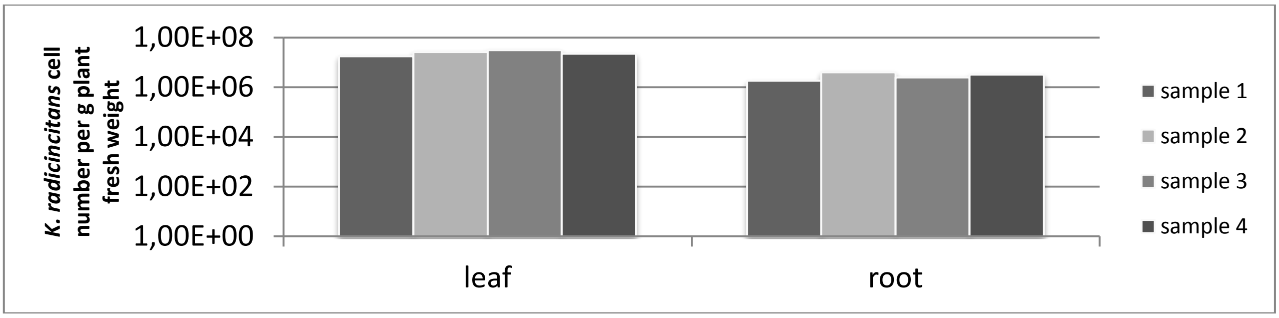

Fig. S8: Colonization of tomato leaf and root tissue by *Kosakonia radicincitans* DSM 16656^{T} determined by qPCR using the target gene calibration curve.

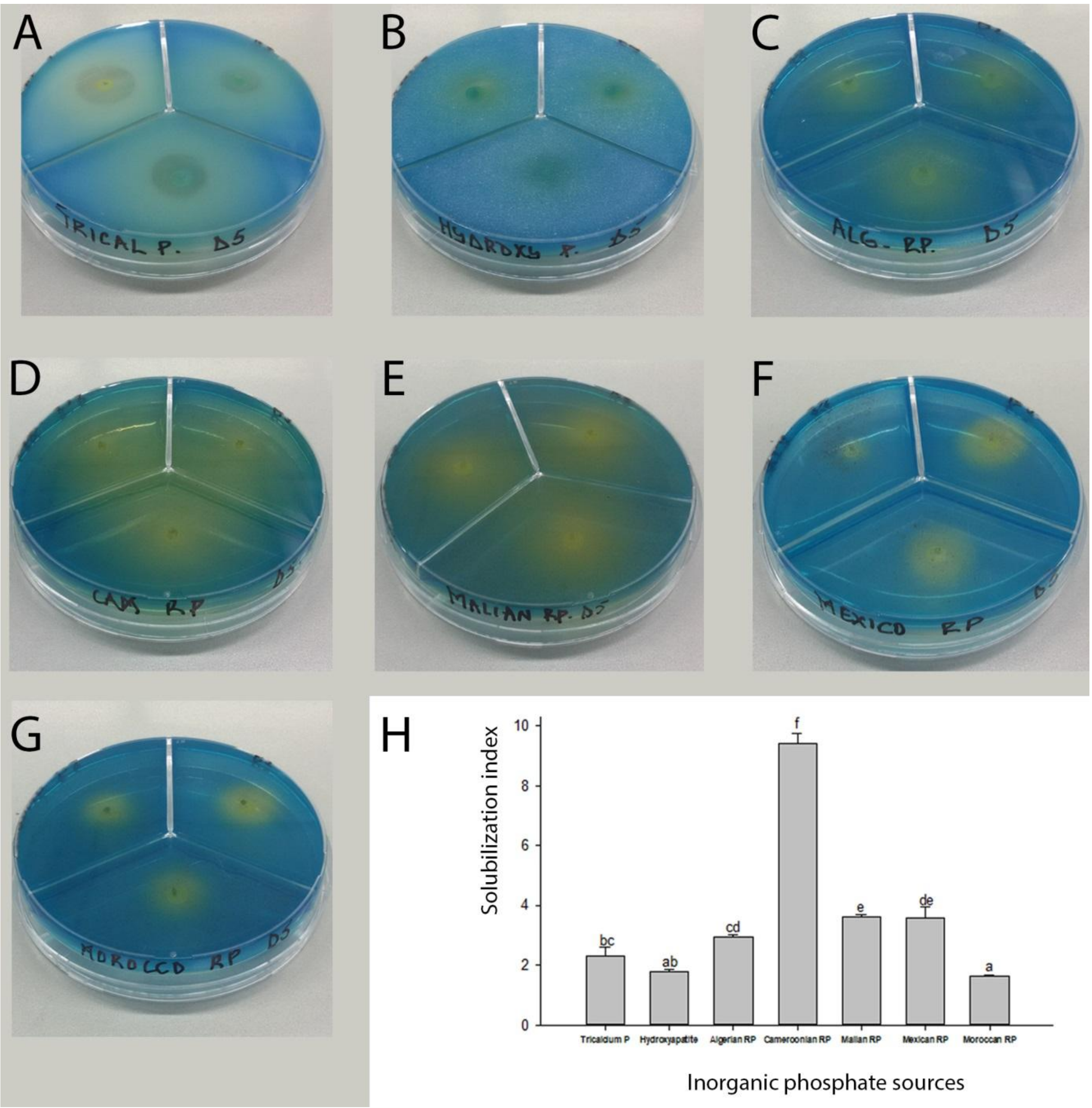

Fig. S9: Ability of *K. radicincitans* DSM 16656^{T} to solubilize inorganic phosphate sources: **(A)** Tricalcium phosphate, **(B)** Hydroxyapatite, and **(C)**-**(G)** Rock phosphate (RP) from different origins: **(C)** Algerian RP, **(D)** Cameroonian RP, **(E)** Malian RP, **(F)** Mexican RP and **(G)** Moroccan RP on NBRIP agar plates. **(H)** Solubilizing index; order of phosphate sources corresponds to order of petri dishes shown in **(A)**-**(G)**; lower case letters above bars indicate significant differences in phosphate solubilization activity ($p < 0.05$) using Tukey test.

Tab. S1: Basic information about *Kosakonia* spp. and closely related strains of other genera, including hosts, sampling sites and references

Taxon	Strain	NCBI accession	16S rRNA gene sequence identity to DSM 16656^T	Host/ place of origin	Sampling site	Reference
K. radicincitans	DSM 16656^T	CP018016	99.8-100%	Wheat, W<br>*Triticum aestivum*<br>phyllosphere	Germany (G)	(1)
K. radicincitans	GXGL-4A	NZ_CP015113	99.9%	Maize, M<br>*Zea mays*<br>root surface	China (C)<br>Guangxi	(2)
K. radicincitans	YD4	JSFC01000001	99.9%	Yerba mate, Ym<br>rhizosphere	Argentina (A)	(3)
K. oryzae	Ola 51^T<br>Ola 50<br>Ola 01	NR_116033<br>EF488758<br>EF488760	99.9%<br>99.9%<br>99.8%	Wild rice, R<br>*Oryza latifolia,*<br>roots	China (C)<br>Guangzhou	(4)
**'Endophytic bacterium'**	Y01	FJ205690	99.9%	Cotton, Co<br>*Gossypium arboretum*<br>roots	China (C)<br>Nanjing	(5)
K. oryzae	22	KC843380	99.9%	desert poplar, P<br>*Populus eutropha*	China (C)<br>Tarim Basin<br>Ugan River	Unpublished
K. radicincitans	ICB22	HQ413268	99.9%<br>91.0% coverage	Sugarcane, S<br>*Saccharum officinarum*<br>stem	Brazil (B)	Unpublished
K. oryzae	SNS4ii	AB975359	99.9%<br>93.9% coverage	Soybean, G<br>*Glycine max*<br>root nodules	Pakistan (P)	Unpublished, AB975359
K. radicincitans	UMEnt01/12	NZ_JDYJ01000000	99.8%	Banana, B<br>*Musa sp.*	Malaysia (Ma)	(6)
K. oryzae	D4	LT799040	99.8%	Not applicable	USA (US)?	Unpublished
E. sp.	BN-St4<br>PB-SRSt	GU459203<br>GU459212	99.8%<br>99.6%	Sugarcane, S<br>*Saccharum officinarum*<br>rhizosphere	Pakistan (P)<br>Punjab<br>Province	(7)
K. oryzae	AB285	HQ706110	99.8%<br>98.3% coverage	Pineapple, A<br>*Ananas comosus*	Brazil (B)<br>Fortaleza	(8)
K. radicincitans	ICB561<br>ICB565<br>ICB 567<br>ICB 573	HM013845<br>HM748090<br>HM748091<br>HQ700329	99.8%<br>91.3-91.6%<br>coverage	Sugarcane, S<br>*Saccharum officinarum*	Brazil (B)	Unpublished
E. sp.	R5-326<br>R5-395<br>R5-424<br>L1-1	JQ659728<br>JQ659764<br>JQ659772<br>JQ659304	99.8%<br>99.7%<br>99.7%<br>99.7%	Purging nut, J<br>*Jatropha curcas*	Singapore (Si)<br>accessions from<br>Indonesia, China<br>and India	(9)
K. radicincitans	159-C<br>172	KP974657<br>KP993224	99.7%<br>99.7% coverage	Sugarcane, S<br>*Saccharum officinarum*<br>roots	Northeast Brazil<br>(B)	Unpublished
E. sp.	NN145S<br>YL34S<br>LA4E<br>GG49E<br>QZ80E<br>LZ84E<br>QZ25S	HQ204285<br>HQ204291<br>HQ204310<br>HQ204300<br>HQ204317<br>HQ204307<br>HQ204288	99.5-99.7%<br>98.2% coverage	Sugarcane, S<br>*Saccharum officinarum*<br>rhizosphere	China (C)<br>Guangxi	(10)<br>(11)
K. arachidis	AW3	AB975353	99.7%<br>45.8% coverage	Wheat, W<br>*Triticum aestivum*<br>soil	Pakistan (P)<br>Rawalpindi	Unpublished
K. oryzae	WL4(1)R<br>S75E<br>S36(1)R<br>S63(1)S	JF513182<br>JF513181<br>JF513180<br>JF513177	98.9-99.6%<br>97.2-99.5%<br>coverage	Salt affected soil	India (I)<br>Karnataka	Unpublished
E. sp.	UYSO10	JF262582	99.6%	Sugarcane, S<br>*Saccharum officinarum*	Uruguay (U)	(12)
K. oryzae	IHB B 6845	KF668469	99.6%<br>98.4% coverage	Agarwood<br>*Aquilaria agallocha*<br>tree core	India (I)<br>Palampu<br>r	Unpublished
E. sp.	NCCP-231	AB610883	99.6%<br>95.7% coverage	Chickpea, C<br>*Cicer arietinum*<br>root nodules	Pakistan (P)<br>Attock	Unpublished

K.oryzae	**NB2**	JX088114	99.6%<br>93.6%coverage	Tomato<br>*Solanum lycopersicum*<br>rhizosphere soil	India (I)<br>Bangalore	Unpublished
Kl.aerogenes	**LSRC164**	JF772083	99.6%	fruit fly<br>*Bactrocera dorsalis*<br>(insect)	China (C)?	Unpublished
K.radicincitans	**256-2**	KP974660	99.5%<br>99.1%coverage	Sugarcane, S<br>*Saccharum officinarum*<br>shoots	Northeast Brazil<br>(B)	Unpublished
K.oryzae	**Fo8A1**	KJ605844	99.5%<br>94.3%coverage	Banana, B<br>*Musa sp.*<br>Surface sterilized root	Taiwan,<br>China (C)	(13)
K.oryzae	**IAC/BECa-086**	KJ670091	99.1%	Sugarcane, S<br>*Saccharum officinarum*<br>roots and stem	Brazil (B)	(14, 15)
K.oryzae	**Lor-MGB-LNK-16**	KC754746	99.1%	rice water weevil<br>(insect)	USA (US)	Unpublished
K.arachidis	**Ah-143**^{T}	NR_116403	98.8%	Groundnut, Ar<br>*Arachis hypogaea*	India (I)<br>Coimbatore	(16)
K.oryziphilus	**REICA_142**<br>**REICA_191**<br>**REICA_084**	JF795013<br>JF795014<br>JF795012	98.6%<br>98.6%<br>98.2%	Rice, R<br>*Oryza sativa*<br>root endosphere	Philippines (Ph)<br>Los Baños	(17)
E.sp.	**FY-07**	NZ_CP012487	98.6%	Oil field	China (C)<br>Jilin	(18)
E.oryzendophyticus	**REICA_211**<br>**REICA_082**<br>**REICA_032**	JF795015<br>JF795011<br>JF795010	98.5%<br>98.4%<br>98.3%	Rice, R<br>*Oryza sativa*	Philippines (Ph)<br>Los Baños,	(19)
E.lignolyticus	D5	CP012871	98.0%	Soil	Malaysia (Ma)	Unpublished
E.lignolyticus	TG1= JD-TRS-7	KJ767522	96.9%	Tea, Te<br>*Camelia sinensis*<br>rhizosphere soil	India (I)<br>Assam	(20)
E.sp.	**DRSBII7**<br>**DRSBII10**	JF514549<br>JF514550	98.5%	Tropical sundew<br>*Drosera burmanii*	India (I)	Unpublished
E.cloacae	**E23**	HM585374	98.5%<br>98.3%coverage	Tomato<br>*Solanum lycopersicum*<br>fruit	Egypt	Unpublished
K.sacchari	**R1**	KF953912	98.4%<br>88.2%coverage	Pigeon pea, Pi<br>*Cajanus cajan*<br>root nodule	India (I)<br>Surat	Unpublished
K.sacchari	**SP1**^{T}	NR_118333	98.4%	Sugarcane, S<br>*Saccharum officinarum*	China (C)<br>Guangxi	(10)
K.sacchari	**G1**	KJ567004	98.4%	Tea tree<br>*Melaleuca*?	China (C)<br>Fuzhou	Unpublished
E.sp.	**R4-368**	NC_021500	98.4%	Purging nut, J<br>*Jatropha curcas*<br>root	Singapore (Si)	(9)
K.oryzae	**KO348**	JZLI01000045	98.3%	Rice, R<br>*Oryza sativa*<br>root	Italia (It)	(21)
K.sacchari	**BO-1**	NZ_CP016337	98.2%	Sweet potato, I<br>*Ipomoea batatas*	Japan (Jp)	(22)
E.sp.	**NN145S**<br>**NN208E**	HQ204285<br>HQ204314	98.5%<br>98.2%coverage	Sugarcane, S<br>*Saccharum officinarum*<br>rhizosphere	China (C)<br>Guangxi	(11)
K.sacchari	**HX148S**<br>**NN143E**<br>**NN144E**<br>**NN208E**	HQ204281<br>HQ204313<br>HQ204314<br>HQ204315	98.1%	Sugarcane, S<br>*Saccharum officinarum*	China (C)<br>Guangxi	(11)
K.sacchari	**ICB101**<br>**ICB105**<br>**ICB118**	HQ413269<br>HQ413270<br>HM748048	97.8-98.1%	Sugarcane, S<br>*Saccharum officinarum*	Brazil (B)	Unpublished
K.pseudosacchari	**JM-387**	NR_135211	98.0%	Maize, M<br>root	USA (US)<br>Tallassee	(23)
K.sacchari	**SVE9**	KF906843	97.8%	Bristle grass<br>*Setaria verticillata*	India (I)	Unpublished

K.sacchari	RSSL	Not available	97.7%	Rice field soil	Sri Lanka (SL)	Unpublished
K.sp.	S29	KX893413	97.5%	Oil palm<br>*Elaeis sp.*<br>leaves	Singapore (Si)	Unpublished
K.cowanii	BCC009	EU629164<br>(24) BCC078	<97.5% with	*Eucalyptus*<br>98.5% coverage	Uruguay (U)	
Kl.variicola	**DX120E**	CP009274	97.9%	Sugarcane, S<br>*Saccharum officinarum*	China (C)<br>Guangxi	(25, 26)
Kl.variicola	**DSM 15968[T]<br>=F2R9[T]**	CP010523	97.9%	Banana, B<br>*Musa sp.*	Mexico (M)<br>Colima<br>(28) roots	(27)
Kl.variicola	**342**	CP000964	97.7%	Maize, M<br>*Zea mays*<br>stems	USA (US)<br>Wisconsin-Madison	(29)
Kl.sp.	**D5A**	JQ277465	96.8%	Tall fescue, F<br>*Festuca arundinacea*<br>rhizosphere soil	China (C)<br>Shandong	(30)
Kl.sp.	**SBP-8**	CP016906	97.9%	Great Millet, So<br>*Sorghum bicolor*,<br>rhizosphere	India (I)<br>Rajasthan	(31)
E.cloacae	**MSR1**	KJ668861	97.6%	Alfalfa, Al<br>*Medicago sativa*	Saudi-Arabia (SA)<br>Al-Ahsaa	(32)
E.cloacae	**SN19**	JQ904624	97.9%	*Teramnus labialis*<br>tropical vine<br>rhizosphere	India (I)	(33)
E.cloacae	**P101**	NZ_CP006580	97.5%	Switchgrass, Pa<br>*Panicum virgatum*	USA (US)<br>Wisconsin	(34)
E.sp.	**DC3**<br>**DC4**	AZXZ01000002<br>AZUB01000018	97.5%<br>97.5%	Yellow yam, Y<br>*Dioscorea cayenensis*	Jamaica (J)	(35)
E.cloacae	**ENHKU01**	NC_018405	97.4%	Pepper (diseased),<br>*Capsicum annuum*	Hong Kong<br>China (C)	(36)
E.sp.	**E20**	NZ_CP012999	97.3%	Rice, R<br>*Oryza sativa*	China (C)<br>Zhejiang	Unpublished
E.sp.	**SST3**	ALNS01000036	97.2%	Sugarcane<br>*Saccharum officinarum*	Jamaica (J)	(37)
E.sp.	**DC1**	AY919308	97.1%	Yellow yam, Y<br>*Dioscorea cayenensis*	Jamaica (J)	(35)
E.cloacae	**GS1**	AJXP01000035	97.0%	Rice, R<br>rhizosphere, root	India (I)<br>Madurai	(38, 39)
Kl.sp.	**LTGPAF-6A**	NZ_CP017450	97.0%	Manna tree<br>*Alhagi sparsifolia*	China (C)<br>Xinjiang	Unpublished
E.sp.	**UCD-UG_FMILLET**	NZ_JRJC01000023	97.0%	Finger Millet<br>*Eleucine coracana*<br>finger surface sterilized roots<br>seeds	Canada (Ca)<br>Planted<br>millet	(40)
E.sp.	**UYSO5**<br>**UYSO6**<br>**UYSO7**<br>**UYSO8**	JF262584<br>JF262585<br>JF262586<br>JF262587	96.6-96.8%	Sugarcane, S<br>*Saccharum officinarum*	Uruguay (U)	(12)
E.sp.	**638**	NC_009436	96.7%	Poplar, P<br>*Populus trichocarpa × P. deltoids*	USA (US)<br>Washington	(41, 42)
E.sp.	**UYSO9**	JF262588	96.4%	Sugarcane, S<br>*Saccharum officinarum*	Uruguay (U)	(12)
E.cloacae	**BN-St2**<br>**LH-St1**<br>**PB-S2**<br>**PB-S1**	GU459201<br>GU459207<br>GU459209<br>GU459208	96.5%<br>96.4%<br>96.2%<br>96.0%	Sugarcane, S<br>*Saccharum officinarum*<br>rhizosphere	Pakistan (P)<br>Punjab Province	(7)
E.cloacae	**PD-P6**	KP259668	97.2%<br>25.5% coverage	Date palm<br>*Phoenix dactylifera*<br>rhizosphere	Oman	(43)
Kluyvera intermedia	CAV1151	NZ_CP011602	98.1%	*Homo sapiens*<br>perirectal	USA (US)	Unpublished

C. sp.	FDAARGOS_156	NZ_CP014030	97.5	*Homo sapiens* stool	USA (US)	Unpublished
C. freundii	CAV1321	NZ_CP011612	97.0	*Homo sapiens* urine/genitourinary	USA (US)	Unpublished
C. amalonaticus	FDAARGOS_165	NZ_CP014070	97.2	*Homo sapiens* stool	USA (US)	Unpublished
C. amalonaticus	FDAARGOS_122	NZ_CP014015	97.1	*Homo sapiens* urine	USA (US)	Unpublished
C. koseri	FDAARGOS_164	LORR01000010	97.1	*Homo sapiens* urine cathether	USA (US)	Unpublished
C. amalonaticus	Y19	NZ_CP011132	96.9	waste-water sludge digester	South Korea	(44)

C = *Citrobacter*, E = *Enterobacter*, K = *Kosakonia*, Kl. = *Klebsiella*; unpublished = according to NCBI

References

1. Remus R, Ruppel S, Jacob H-J, Hecht-Buchholz C, Merbach W. 2000. Colonization behaviour of two enterobacterial strains on cereals. Biology and Fertility of Soils 30:550-557.
2. Li Q-J, Cheng J-J, Sun S-X, Chen Y-P. 2016. Isolation, identification and characterization of associative nitrogen-fixing endophytic bacterium *Kosakonia radicincitans* GXGL-4A in maize. Microbiology China 43:2456-2463.
3. Bergottini VM, Filippidou S, Junier T, Johnson S, Chain PS, Otegui MB, Zapata PD, Junier P. 2015. Genome sequence of *Kosakonia radicincitans* strain YD4, a plant growth-promoting rhizobacterium isolated from Yerba Mate (*Ilex paraguariensis St. Hill.*). Genome Announc 3.
4. Peng G, Zhang W, Luo H, Xie H, Lai W, Tan Z. 2009. Enterobacter oryzae sp. nov., a nitrogen-fixing bacterium isolated from the wild rice species Oryza latifolia. International Journal of Systematic and Evolutionary Microbiology 59:1650-1655.
5. Li CH, Zhao MW, Tang CM, Li SP. 2010. Population dynamics and identification of endophytic bacteria antagonistic toward plant-pathogenic fungi in cotton root. Microb Ecol 59:344-56.
6. Suhaimi NS, Yap KP, Ajam N, Thong KL. 2014. Genome sequence of *Kosakonia radicincitans UMEnt01/12*, a bacterium associated with bacterial wilt diseased banana plant. FEMS Microbiol Lett 358:11-3.
7. Mehnaz S, Baig DN, Lazarovits G. 2010. Genetic and phenotypic diversity of plant growth promoting rhizobacteria isolated from sugarcane plants growing in pakistan. J Microbiol Biotechnol 20:1614-23.
8. Weber OB, Videira SS, Simoes de Araujo JL. 2013. Identification of culturable endophytes in Champaka pineapple grown in an organic system. Afr J Agric Res 8:3422-3430.
9. Madhaiyan M, Peng N, Te NS, Hsin IC, Lin C, Lin F, Reddy C, Yan H, Ji L. 2013. Improvement of plant growth and seed yield in Jatropha curcas by a novel nitrogen-fixing root associated Enterobacter species. Biotechnol Biofuels 6:140.
10. Zhu B, Zhou Q, Lin L, Hu C, Shen P, Yang L, An Q, Xie G, Li Y. 2013. Enterobacter sacchari sp. nov., a nitrogen-fixing bacterium associated with sugar cane (Saccharum officinarum L.). Int J Syst Evol Microbiol 63:2577-82.
11. Lin L, Li Z, Hu C, Zhang X, Chang S, Yang L, Li Y, An Q. 2012. Plant growth-promoting nitrogen-fixing enterobacteria are in association with sugarcane plants growing in Guangxi, China. Microbes Environ 27:391-8.
12. Taulé C, Mareque C, Barlocco C, Hackembruch F, Reis VM, Sicardi M, Battistoni F. 2012. The contribution of nitrogen fixation to sugarcane (Saccharum officinarum L.), and the identification and characterization of part of the associated diazotrophic bacterial community. Plant and Soil 356:35-49.

13. Ho Y-N, Chiang H-M, Chao C-P, Su C-C, Hsu H-F, Guo C-t, Hsieh J-L, Huang C-C. 2015. In planta biocontrol of soilborne Fusarium wilt of banana through a plant endophytic bacterium, Burkholderia cenocepacia 869T2. Plant and Soil 387:295-306.
14. Magnani GS, Didonet CM, Cruz LM, Picheth CF, Pedrosa FO, Souza EM. 2010. Diversity of endophytic bacteria in Brazilian sugarcane. Genet Mol Res 9:250-8.
15. Marcos FCC, Iório RdPF, Silveira APDd, Ribeiro RV, Machado EC, Lagôa AMMdA. 2016. Endophytic bacteria affect sugarcane physiology without changing plant growth. Bragantia 75:1-9.
16. Madhaiyan M, Poonguzhali S, Lee JS, Saravanan VS, Lee KC, Santhanakrishnan P. 2010. Enterobacter arachidis sp. nov., a plant-growth-promoting diazotrophic bacterium isolated from rhizosphere soil of groundnut. Int J Syst Evol Microbiol 60:1559-64.
17. Hardoim PR, van Overbeek LS, Berg G, Pirttila AM, Compant S, Campisano A, Doring M, Sessitsch A. 2015. The hidden world within plants: ecological and evolutionary considerations for defining functioning of microbial endophytes. Microbiol Mol Biol Rev 79:293-320.
18. Ji K, Wang W, Zeng B, Chen S, Zhao Q, Chen Y, Li G, Ma T. 2016. Bacterial cellulose synthesis mechanism of facultative anaerobe *Enterobacter sp. FY-07*. Sci Rep 6:21863.
19. Hardoim PR, Nazir R, Sessitsch A, Elhottová D, Korenblum E, van Overbeek LS, van Elsas JD. 2013. The new species Enterobacter oryziphilus sp. nov. and Enterobacter oryzendophyticus sp. nov. are key inhabitants of the endosphere of rice. BMC Microbiol 13:164.
20. Dutta J, Handique PJ, Thakur D. 2015. Assessment of culturable tea rhizobacteria isolated from tea estates of Assam, India for growth promotion in commercial tea cultivars. Front Microbiol 6:1252.
21. Meng X, Bertani I, Abbruscato P, Piffanelli P, Licastro D, Wang C, Venturi V. 2015. Draft Genome Sequence of Rice Endophyte-Associated Isolate Kosakonia oryzae KO348. Genome Announc 3.
22. Shinjo R, Uesaka K, Ihara K, Loshakova K, Mizuno Y, Yano K, Tanaka A. 2016. Complete Genome Sequence of Kosakonia sacchari Strain BO-1, an Endophytic Diazotroph Isolated from a Sweet Potato. Genome Announc 4.
23. Kampfer P, McInroy JA, Doijad S, Chakraborty T, Glaeser SP. 2016. *Kosakonia pseudosacchari sp nov*., an endophyte of *Zea mays*. Systematic and Applied Microbiology 39:1-7.
24. Brady CL, Venter SN, Cleenwerck I, Engelbeen K, de Vos P, Wingfield MJ, Telechea N, Coutinho TA. 2009. Isolation of Enterobacter cowanii from Eucalyptus showing symptoms of bacterial blight and dieback in Uruguay. Lett Appl Microbiol 49:461-5.
25. Wei CY, Xing YX, Lin L, Yang LT, Li YR, Hu CJ. 2014. [Growth-promoting effect of inoculating Klebsiella variicola DX120E on different sugarcane cultivars]. Ying Yong Sheng Tai Xue Bao 25:2085-92.
26. Lin L, Wei C, Chen M, Wang H, Li Y, Li Y, Yang L, An Q. 2015. Complete genome sequence of endophytic nitrogen-fixing *Klebsiella variicola strain DX120E*. Stand Genomic Sci 10:22.
27. Rosenblueth M, Martinez L, Silva J, Martinez-Romero E. 2004. Klebsiella variicola, a novel species with clinical and plant-associated isolates. Syst Appl Microbiol 27:27-35.
28. Chen M, Li Y, Li S, Tang L, Zheng J, An Q. 2016. Genomic identification of nitrogen-fixing *Klebsiella variicola*, *K. pneumoniae* and *K. quasipneumoniae*. J Basic Microbiol 56:78-84.
29. Dong Y, Glasner JD, Blattner FR, Triplett EW. 2001. Genomic interspecies microarray hybridization: rapid discovery of three thousand genes in the maize endophyte, *Klebsiella pneumoniae 342*, by microarray hybridization with *Escherichia coli K-12* open reading frames. Appl Environ Microbiol67:1911-21.
30. Liu W, Wang Q, Hou J, Tu C, Luo Y, Christie P. 2016. Whole genome analysis of halotolerant and alkalotolerant plant growth-promoting rhizobacterium *Klebsiella sp. D5A*. Sci Rep 6:26710.
31. Singh RP, Jha P, Jha PN. 2015. The plant-growth-promoting bacterium Klebsiella sp. SBP-8 confers induced systemic tolerance in wheat (Triticum aestivum) under salt stress. J Plant Physiol 184:57-67.

32. Khalifa AY, Alsyeeh AM, Almalki MA, Saleh FA. 2016. Characterization of the plant growth promoting bacterium, Enterobacter cloacae MSR1, isolated from roots of non-nodulating Medicago sativa. Saudi J Biol Sci 23:79-86.
33. Bose A, Kher MM, Nataraj M, Keharia H. 2016. Phytostimulatory effect of indole-3-acetic acid by *Enterobacter cloacae SN19* isolated from *Teramnus labialis* (L. f.) spreng rhizosphere. Biocatalysis and Agricultural Biotechnology 6:128-137.
34. Humann JL, Wildung M, Pouchnik D, Bates AA, Drew JC, Zipperer UN, Triplett EW, Main D, Schroeder BK. 2014. Complete genome of the switchgrass endophyte *Enterobacter clocace P101*. Stand Genomic Sci 9:726-34.
35. Gan HM, Triassi AJ, Wheatley MS, Savka MA, Hudson AO. 2014. High-quality draft whole-genome sequences of three strains of *Enterobacter* isolated from Jamaican *Dioscorea cayenensis* (Yellow Yam). Genome Announc 2.
36. Liu WY, Chung KM, Wong CF, Jiang JW, Hui RK, Leung FC. 2012. Complete genome sequence of the endophytic *Enterobacter cloacae subsp. cloacae* strain *ENHKU01*. J Bacteriol 194:5965.
37. Gan HM, McGroty SE, Chew TH, Chan KG, Buckley LJ, Savka MA, Hudson AO. 2012. Whole-genome sequence of *Enterobacter sp. strain SST3,* an endophyte isolated from Jamaican sugarcane (*Saccharum sp.*) stalk tissue. J Bacteriol 194:5981-2.
38. Shankar M, Ponraj P, Ilakkiam D, Gunasekaran P. 2011. Root colonization of a rice growth promoting strain of Enterobacter cloacae. J Basic Microbiol 51:523-30.
39. Shankar M, Ponraj P, Ilakiam D, Rajendhran J, Gunasekaran P. 2012. Genome sequence of the plant growth-promoting bacterium Enterobacter cloacae GS1. J Bacteriol 194:4479.
40. Ettinger CL, Mousa WM, Raizada MN, Eisen JA. 2015. Draft Genome Sequence of Enterobacter sp. Strain UCD-UG_FMILLET (Phylum Proteobacteria). Genome Announc 3.
41. Taghavi S, van der Lelie D, Hoffman A, Zhang YB, Walla MD, Vangronsveld J, Newman L, Monchy S. 2010. Genome sequence of the plant growth promoting endophytic bacterium *Enterobacter sp*. 638. PLoS Genet 6:e1000943.
42. Taghavi S, Garafola C, Monchy S, Newman L, Hoffman A, Weyens N, Barac T, Vangronsveld J, van der Lelie D. 2009. Genome survey and characterization of endophytic bacteria exhibiting a beneficial effect on growth and development of poplar trees. Appl Environ Microbiol 75:748-57.
43. Yaish MW, Antony I, Glick BR. 2015. Isolation and characterization of endophytic plant growth-promoting bacteria from date palm tree (Phoenix dactylifera L.) and their potential role in salinity tolerance. Antonie Van Leeuwenhoek 107:1519-32.
44. Ainala SK, Ashok S, Ko Y, Park S. 2013. Glycerol assimilation and production of 1,3-propanediol by Citrobacter amalonaticus Y19. Appl Microbiol Biotechnol 97:5001-11.

Tab. S2: Plant growth-promoting effects of *Kosakonia radicincitans* DSM16656^T

Year	G*/F*	Crop/ plant	Cultivar/accession	Criterion	Efficiency** (increase compared to respective control)	Reference
**1987**	F	Wheat (*Triticum aestivum* L.)	Taras	grain yield	+5%	(1)
**1988**	F		Alcedo		+13%	
**1989**	F		Regina		+8%	
**1990**	F		Regina		+8%	
**1995**	F		Bussard		+21%	
**1995**	F		Greif		+23%	
**1995**	F		Ramiro		+3%	
**1989, 1990**	F	Pea (*Pisum sativum* L.)	Grapis	dry matter	+48%	(2)
				seed yield	+19%, +29%	
**1990**	F	Maize (*Zea mays* L.)	Bekenova	shoot dry matter	+4%	(3)
				grain yield	+9%	
**1991**	F			shoot dry matter	+7%	
				grain yield	+15%	
**1992**	F			shoot dry matter	+3%	
				grain yield	+8%	
**2006**	G	Kohlrabi (*Brassica oleracea* var. *gongylodes* L.)	RZ Eder	root dry matter	+47%	(4)
				leaf dry matter	+36%	
				tuber dry matter	+37%	
**2007**	F	Oilseed rape (*Brassica napus* L.)		shoot biomass	+5%, +12%	(5)
**2010**	F	Radish (*R. sativus var. sativus* L.)	Rondar	tuber yield	+23%	(6)
				leaf yield	+20%	
**2011**	F			tuber yield	+14%	
				leaf yield	+23%	
**2012**	G	Thale cress (*Arabidopsis thaliana* L.)	Columbia	seed weight	+ up to 30%	(7)
				rosette diameter	+ approx. 10%	
**2012**	G	Tomato (*Solanum lycopersicum* L.)	Vanessa	root dry matter	+120%, +180%	(8)
				shoot dry matter	+140%, +150%	
				stalk length	+27%, +29%	

G*: glasshouse experiment F*: field experiment, **depending on concentration of inoculant

1. Remus R, Ruppel S, Jacob HJ, Hecht-Buchholz C, Merbach W. 2000. Colonization behaviour of two enterobacterial strains on cereals. Biol Fert Soils 30:550-557.
2. Höflich G, Wiehe W, Kühn G. 1994. Plant growth stimulation by inoculation with symbiotic and associative rhizosphere microorganisms. Experientia 50:897-905.
3. Ruppel S. 2000. Bedeutung der rhizosphären- und endophytischen Bakterien für die Pflanzenernährung. Arch Agron Soil Sci 45:329-341.
4. Ruppel S, Rühlmann J, Merbach W. 2006. Quantification and localization of bacteria in plant tissues using quantitative real-time PCR and online emission fingerprinting. Plant Soil 286.
5. Krey T, Caus M, Baum C, Ruppel S, Eichler-Lobermann B. 2011. Interactive effects of plant growth-promoting rhizobacteria and organic fertilization on P nutrition of *Zea mays* L. and *Brassica napus* L. J Plant Nutr Soil Sc 174:602-613.
6. Berger B, Wiesner M, Brock AK, Schreiner M, Ruppel S. 2015. *K. radicincitans*, a beneficial bacteria that promotes radish growth under field conditions. Agron Sustain Dev 35:1521-1528.
7. Brock AK, Berger B, Mewis I, Ruppel S. 2013. Impact of the PGPB *Enterobacter radicincitans* DSM 16656 on growth, glucosinolate profile, and immune responses of *Arabidopsis thaliana*. Microb Ecol 65:661-670.
8. Berger B, Brock AK, Ruppel S. 2013. Nitrogen supply influences plant growth and transcriptional responses induced by *Enterobacter radicincitans* in *Solanum lycopersicum*. Plant Soil 370:641-65

The closely related *Kosakonia arachidis* Ah-143^T offers a similar potential like *K. radicincitans* DSM 16656^T. *K. arachidis* Ah-143^T inoculated mustard, tomato or rice seeds increased their root length by 61.1, 21.6 and 10.4 %, respectively. Sugarcane seedlings that were inoculated with *Kosakonia sacchari* SP1^T showed higher dry weights and nitrogen contents than those of non-inoculated controls plants (Chen et al., 2014). Though several tree species have been reported to host *Kosakonia* and closely related strains only one tree species was described to benefit from the inoculation with PGPB. *Enterobacter sp.* 638 increased the growth index of different poplar varieties by up to 40% compared to their respective controls (Taghavi et al., 2010). The endophytic bacterium Y01 (FJ205690) from cotton (*Gossypium arboreum*), sharing 99.9% of 16S rRNA sequence with DSM 16656^T, was shown to be the only strain out of 39 isolated ones – chosen by antagonism to fungal pathogens – that also increased shoot length. *Enterobacter sp*. NN145S and *Kosakonia sacchari* NN143E isolated from rhizosphere soil and surface-sterilized roots, respectively, of the same ROC22 plant were used to inoculate micropropagated sugarcane plantlets. Both strains increased the biomass and nitrogen content of the sugarcane seedlings (Lin et al., 2012).

References

Chen MY, Zhu B, Lin L, Yang LT, Li YR & An QL (2014) Complete genome sequence of *Kosakonia sacchari type strain SP1*(T). *Stand Genomic Sci* **9**.

Lin L, Li Z, Hu C, Zhang X, Chang S, Yang L, Li Y & An Q (2012) Plant growth-promoting nitrogen-fixing enterobacteria are in association with sugarcane plants growing in Guangxi, China. *Microbes Environ* **27**: 391-398.

Taghavi S, van der Lelie D, Hoffman A, Zhang YB, Walla MD, Vangronsveld J, Newman L & Monchy S (2010) Genome sequence of the plant growth promoting endophytic bacterium *Enterobacter sp*. 638. *PLoS Genet* **6**: e1000943.

Tab. S3: Number of annotated genes of *Kosakonia radicincitans* DSM16656^T and expression profile from strain-specific microarray experiment in which liquid culture of DSM16656^T was exposed to tomato root exudates

**Total Genes**	**6058**	**Chr.: 5680, L.pl.: 359, S.pl.: 19**	
CDS	5839	Chr.: 5465, L.pl.: 355, S.pl.: 19	
rRNA	22	Chr.: 5S:8 ❙ 16S:7 ❙ 23S: 7	
tRNA	83	Chr.: 83	
other RNAs	114	Chr.: 110, L.pl.: 4	
**CDS**	**5839**	**Chr.: 5465, L.pl.: 355, S.pl.: 19**	
**PROKKA annotated**	**4756**	**Chr.: 4602, L.pl.: 151, S.pl.: 3**	
PROKKA hypothetical proteins	1083	Chr.: 863, L.pl.: 204, S.pl.: 16	
Interpro (GO) annotated	3828	Chr.: 3723 , L.pl.: 102, S.pl.: 3	
Koala (KEGG) annotated	4337	Chr.: 4056, L.pl.: 266, S.pl.: 15	
GO+KEGG	5081	Chr.: 4781, L.pl.: 283, S.pl.: 17	
PROKKA+GO+KEGG	5469	Chr.: 5152, L.pl.: 300, S.pl.: 17	
CDS represented on Microarray	5512	Chr.: 5243, L.pl.: 269	
**PROKKA hypothetical proteins**	**1083**	Chr.: 863, L.pl.: 204, S.pl.: 16	
Inhouse pipeline combining PROKKA, GO and KEGG: missing function	370	Chr.: 313, L.pl.: 55, S.pl.: 2	
		**Significant expression changes**	
		**number of genes**	**percentage**
**CDS with expression profile**	**5512**	***2959 (pV=0.05): Chr.: 2860, L.pl.: 99**	**of 2959 genes**
up-regulated	2483	*1470 (pV=0.05): Chr.: 1414, L.pl.: 56	50% up
down-regulated	3029	*1489 (pV=0.05) : Chr.: 1446, L.pl.: 43	50% down
FC > =❙2❙ (FC 2)	1658	***1526** (pV=0.05) : Chr.: 1481, L.pl.: 45	**of 1526 genes**
up-regulated (FC 2)	880	*843 (pV=0.05) : Chr.: 820, L.pl.: 23	55% up
down-regulated (FC 2)	778	*683 (pV=0.05) : Chr.: 661, L.pl.: 22	45% down
FC > =|1,5| (FC 1,5)	2791	***2332** (pV=0.05) : Chr.: 2260, L.pl.: 72	**of 2332 genes**
up-regulated (FC 1,5)	1355	*1197 (pV= 0.05) : Chr.: 1156, L.pl.: 41	51% up
down-regulated (FC 1,5)	1436	*1135 (pV=0.05) : Chr.: 1104, L.pl.: 31	49% down
Motility & Chemotaxis (FC 1,5)	111 (of 214)	***96** (pV=0.05) : Chr.: 93, L.pl.: 3	**of 96 genes**
up-regulated (FC 1,5)	87	*83 (pV=0.05) : Chr.: 80, L.pl.: 3	**86% up**
down-regulated (FC 1,5)	24	*13 (pV=0.05) : Chr.: 13	**14% down**

CDS = coding sequence, Chr. = chromosome, L.pl. = large plasmid, S.pl = small plasmid, FC = fold change, pV = p-value,
*number of genes with significant expression changes, **percentage of genes significantly up-regulated and down-regulated, respectively

Tab. S4: Amino acid sequence identity of T6SS.3 genes of *Kosakonia radicincitans* DSM 16656^{T} to best hits of other bacterial taxa performing BLASTp searches

gene	worst hit within *K. radicincitans*	best other hit
clpV	90.7	69.5
tssM	87.6	56.7
tssL	95.6	58.6
tssK	91.4	73.2
tssJ	90.0	71.4
tssA	90.0	61.8
PAAR	97.0	93.9
tagK1	94.6	55.3
vgrG	86.5	60.4
tssG	92.7	67.8
tssF	93.7	63.4
tssE	80.0	52.9
*hcp**	93.1	67.5
tssC	96.8	88.6
tssB	96.3	85.8
**average**	**91.7**	**68.5**

Tab. S5: Supportive information to **Figure 4B**: Each box represents the exact number of genes shared between *Kosakonia radicincitans* DSM 16656^{T} and another bacterial strain per gene cluster of particular interest

strain	KFS.1a	KFS.1b	KFS.2	T6SS.1	T6SS.2	T6SS.3	*xerC*	NAR	NIF	ANF	PHO	PST	*ipdC*	GAB	*dcyD*	BUD	CYS	OPU	PRO	ENT	FEP	FHU	*pqqE*	la-pla	sm-pla
DSM 16656	71	14	55	14	19	13	6	9	20	7	8	5	1	4	1	3	9	10	4	9	6	12	1	355	19
Ola 51	70	14	55	14	16	13	2	8	20	7	8	5	1	4	1	3	9	10	4	9	6	12	0	0	0
GXGL-4A	71	14	55	10	19	13	4	8	20	7	8	5	1	4	1	3	9	10	4	9	6	12	0	4	1
YD4	71	14	55	14	17	13	1	8	20	7	8	5	1	4	1	3	9	10	4	9	6	12	0	1	0
UMEnt01/12	68	14	53	13	18	12	5	8	20	7	8	5	1	4	1	3	9	10	4	9	6	11	0	14	0
REICA_142	63	14	5	13	16	0	1	8	20	0	8	5	1	3	1	3	9	0	3	8	5	6	0	10	1
FY-07	63	14	3	0	17	0	2	8	20	0	8	4	1	2	1	3	9	10	4	8	5	9	0	2	7
REICA_082	64	14	3	0	18	0	1	8	20	0	8	4	1	2	1	3	9	9	4	8	4	9	0	2	0
SP1	68	14	26	14	17	0	2	8	20	0	7	5	1	3	1	3	9	4	3	8	5	10	0	5	0
R4-368	68	14	52	14	17	0	2	8	20	0	7	5	1	3	1	3	9	4	3	8	4	10	0	12	1
KO348	67	14	6	4	18	0	1	8	20	0	7	5	1	3	1	3	9	4	3	8	4	10	0	3	0
BO-1	68	14	4	0	17	0	1	8	20	0	7	5	1	3	1	3	9	4	3	8	5	9	0	7	0
SBP-8	59	14	4	4	16	0	1	7	0	0	5	4	1	3	1	3	9	4	3	7	5	7	0	3	0
DX120E	25	0	4	14	0	0	3	7	20	0	8	4	1	3	1	3	9	4	3	7	5	8	0	36	1
DSM 15968	25	0	4	14	0	0	1	7	20	0	8	4	1	3	1	3	9	4	3	7	5	8	0	33	0
342	25	0	4	13	0	0	2	7	20	0	8	4	1	3	1	3	9	4	3	7	5	7	0	49	0
D5A	25	0	4	13	0	0	3	7	20	0	8	4	1	3	1	3	9	4	3	7	5	8	0	34	0
P101	61	14	40	14	18	0	1	7	0	0	6	4	1	3	1	3	9	4	3	7	5	8	0	11	1
DC3	58	14	4	14	17	0	2	7	0	0	6	4	1	3	1	3	9	4	3	7	5	7	0	15	0
DC4	58	14	49	14	17	0	3	7	0	0	6	4	1	3	1	3	9	4	3	7	5	7	0	17	0
ENHKU01	57	14	2	14	16	0	1	7	0	0	5	4	1	3	1	3	9	4	3	6	5	8	0	5	0
SST3	56	14	4	4	16	0	2	7	0	0	6	4	1	3	1	3	9	4	3	7	4	7	0	8	1
DC1	56	14	4	13	17	0	1	7	0	0	6	4	1	3	1	3	9	4	3	6	5	7	0	10	1
GS1	59	14	2	5	7	0	1	7	0	0	5	4	1	3	1	3	7	4	1	3	3	7	0	1	0
638	63	14	0	6	0	0	2	7	0	0	6	4	1	3	1	3	8	4	3	7	4	9	0	12	1
UCD-UG_FMILLET	59	14	3	14	16	0	1	7	0	0	5	4	1	3	1	3	9	4	3	7	4	8	0	18	1
FDAARGOS_156	61	13	11	14	0	0	2	7	0	0	6	4	1	2	1	0	8	0	3	7	4	10	0	30	0
CAV1321	62	14	2	1	0	0	2	7	0	0	6	4	1	2	1	0	8	0	3	7	5	9	1	254	1
FDAARGOS_165	58	14	2	6	3	0	3	6	0	0	6	4	0	2	1	0	7	4	3	6	5	6	0	32	1
FDAARGOS_164	58	14	14	8	1	0	1	7	0	0	5	4	1	2	1	0	8	4	3	6	6	9	0	28	1
FDAARGOS_122	62	14	0	0	0	0	1	7	0	1	6	4	1	2	1	0	8	4	3	6	5	5	0	31	0
Y19	61	14	0	2	2	3	4	7	0	0	6	4	1	2	1	0	8	4	3	7	5	7	0	230	1
F113	40	8	22	9	14	4	1	7	1	0	3	3	0	2	1	1	7	6	2	2	0	3	0	20	0
3841	25	2	12	0	0	0	1	2	8	1	4	4	0	2	0	1	4	2	2	2	0	5	0	23	1
FZB42	20	2	11	0	0	0	0	5	1	0	3	3	0	2	0	3	4	4	2	5	2	1	0	8	0

Tab. S6: Percentage of chromosomal and large plasmid genes of *Kosakonia radicincitans* DSM 16656[T] with KEGG annotation contributing to metabolic pathways

Pathway (KEGG terminology)	chromosome	large plasmid
Metabolic pathways	696 genes	57 genes (24 of them = 42% are missing on chromosome)
Microbial metabolism in diverse environments	33%	39%
Carbon metabolism	16%	21%
Methane metabolism	5%	**14%**
ABC transporters	**28%**	14%
Quorum sensing	8%	12%
Oxidative phosphorylation	6%	9%
Flagellar assembly	5%	5%
Bacterial secretion system	4%	2%
Biofilm formation - *Escherichia coli*	4%	4%
Propanoate metabolism	4%	**18%**
Porphyrin and chlorophyll metabolism	5%	**9%**
Glycerolipid metabolism	2%	**11%**
Glycerophospholipid metabolism	4%	5%
Nitrogen metabolism	4%	2%
Bacterial chemotaxis	3%	5%
Chloroalkane and chloroalkene degradation	1%	5%
Degradation of aromatic compounds	1%	5%

4337 genes of *Kosakonia radicincitans* DSM16656[T] were KEGG annotated, 3070 of them were considered in a KEGG pathway search, and 720 different KEGG identifiers were assigned to pathways.

MATERIAL and METHODS

(including details such as primer sequences and links to applied computer programs)

Characterization of phosphate solubilizing ability of DSM 16656^T on plate assay

The ability of *K. radicincitans* DSM 16656^T to solubilize different inorganic phosphate sources (tricalcium phosphate, hydroxyapatite, Algerian rock phosphate (RP), Cameroonian RP, Malian RP, Mexican RP and Moroccan RP) was assessed on modified NBRIP (National Botanical Research Institute's Phosphate growth medium) plates (Nautiyal 1999). NBRIP medium was prepared with 20 g glucose, 5 g $MgCl_2 \cdot 6H_2O$, 0.25 g $MgSO_4 \cdot 7H_2O$, 0.2 g KCl, 0.1 g $(NH_4)_2SO_4$, one of the abovementioned phosphate types at 5 g, 0.5% Bromocresol Green (BCG), and 15 g agar per L, and pH adjusted to 7.5. The BCG stock solution was prepared by dissolving 5 g BCG in 100 mL of 70% ethanol and pH adjusted to 6.5 with 1M KOH (Fankem et al., 2014). All phosphate sources were washed prior to use to remove soluble fractions by drenching with warm water for 1 h and rinsing. Afterwards, phosphate sources were soaked for 24 h before rinsing. The process was repeated twice. The phosphate sources were dried at 60°C, and homogenized before use. A single bacterial colony was grown over night in 50 mL in nutrient broth (Standard nutrient broth I, Carl Roth, Germany) at 28°C. The OD_{620} was adjusted to 0.2 and 10 µL of bacterial suspension were spotted on three compartmented Petri dishes for each phosphate source. Plates were incubated at 28°C for 7 days and the extent of phosphate solubilization was assessed. The index of solubilization was used as an indicator for the isolate efficiency: IS = (Colony diameter + diameter of halo zone)/ Colony diameter (Fankem et al., 2014).

Bacterial transformation and cultivation for Confocal Laser Scanning Microcopy (CLSM)

For plant colonization studies, electro-competent cells of *K. radicincitans* DSM 16656^T were transformed with enhanced green fluorescent protein (eGFP)-containing plasmid pMP4655 (Bloemberg et al., 2000; Lagendijk et al., 2010). DSM 16656^T cells were made electro competent using a standard protocol (Gonzales et al., 2013). Transformation of DSM 16656^T with pMP4655 was performed using a Micro Pulser (Biorad), and cells were regenerated in 1 mL Super Optimal Broth (SOB) plus 20 mM glucose for 1 h and grown over night on LB supplemented with gentamycin (150 µL/mL) at 30°C. Plasmid integration was shown by colony-PCR using primers MC EG-f (GTGAGCAAGGGCGAGGAGCTG) and MC EG-r (CTTGTACAGCTCGTCCATGCCG) for pMP4655 eGFP amplification. PCR was performed with Dream Taq Polymerase (Qiagen) at 60°C annealing temperature with an elongation time of 40 s for 30 cycles in a 20 µL reaction. Single colonies of DSM 16656^T expressing eGFP grown on Luria-Bertani agar plus gentamycin (150 µg/mL) were cultured overnight in standard nutrient broth (Merck). The cells were pelleted by centrifugation, washed twice in sterile ultrapure water, re-suspended in sterile ultrapure water to give an OD_{620} of 0.2 (corresponding to 10^9 cfu mL^{-1}) and finally further diluted to a concentration of 10^7 cfu mL^{-1}.

Plant cultivation and inoculation of eGFP expressing DSM 16656^T for CLSM

Arabidopsis thaliana accession Col-0 and *S. lycopersicum* cv. Micro-Tom were raised as *in vitro* cultures. Seeds were surface-sterilized with a solution containing 5% NaOCl and 0.5% Tween 20,

rinsed several times in sterile water, dried and incubated at 4°C for 3 days for stratification on sterile plates containing 1/2-strength Murashige-Skoog (Duchefa) medium and afterwards grown under short day conditions (8 h photoperiod at 22°C and 40-60% relative humidity) in a vertical position for two weeks. Plants were transferred to 11 mL sterile ultrapure water and kept for 24 h to adjust to the changed conditions before 10^5 bacterial cells were added to each plant. Water levels were adjusted on a daily basis to account for evaporation and plant-based water losses. Bacterial root colonization was monitored after 6 days. Roots were gently washed in sterile water and fluorescence was recorded with a Zeiss LSM 510 META laser scanning confocal microscope (Carl Zeiss Jena GmbH). Bacterial eGFP fluorescence signals were captured using argon laser excitation at 488 nm (BP505-550 180 filter, Plan Apo 63/1.4 oil lens), and roots were captured using bright field settings.

TEM Microscopy

Conventional negative contrast staining was used for electron microscopic investigations. Cells of *K. radicincitans* DSM 16656^T were taken from semi solid (0.5%) agar and placed in 0.05 M saline solution. One drop of this bacterial solution was applied to Pioloform-carbon-coated, 400-mesh copper grids (Plano GmbH) for 10 min, fixed with 2.5% aqueous glutaraldehyde solution for 1 min, stained with 2.5% uranyl acetate solution for 1 min, and examined by transmission electron microscopy using a JEM-1400 Plus (JEOL) at an acceleration voltage of 120 kV.

Gene annotation and functional annotation

Genome annotation was derived from PROKKA (http://www.vicbioinformatics.com/software.prokka.shtml), RNAmmer (http://www.cbs.dtu.dk/services/RNAmmer/) and ARAGORN (http://130.235.46.10/ARAGORN/). Functional annotation was received from the SEED-based "Rapid Annotations using Subsystems Technology" (RAST) tool from the RAST server (http://rast.nmpdr.org) and pathway classification from "KEGG Orthology And Links Annotation" tool (BlastKOALA, http://www.kegg.jp/blastkoala/).

Genome comparison

The genome sequences of 31 closely related and three more distantly related PGPB (*Pseudomonas fluorescens* F113, *Rhizobium leguminosarum bv. viciae* 3841, *Bacillus velezensis* FZB42) were compared to *K. radicincitans* DSM 16656^T. The phylogenetic distance was calculated according to the 16S rRNA gene sequence after running BLASTn against NCBI and RDP, doing a Muscle Alignment and building a bootstrap supporting NJ-tree (Geneious 8.1.9, Genetic Distance Model: Jukes-Cantor, 10,000 replicates) for the candidate strains. Another tree was generated for the concatenated amino acid sequence of four phylogenetic markers (AtpD, GyrB, InfB, RecA). The nucleotide and amino acid sequences for the whole genomes were retrieved from NCBI. To obtain conserved genomic regions a MAUVE alignment was done for each phylogenetic group: *K. radicincitan*s (KORA), *Kosakonia sacchari* (KOSA), *Enterobacter* (ENTERO) and *Klebsiella variicola* (KLEVA) with our strain as reference.

Furthermore, to detect orthologous and paralogous genes and gene clusters BLASTp+ and an in-house pipeline were applied, considering the reciprocal best hit algorithm (RBH) and scoring, as well

as the conserved regions. The resulting clusters were divided into the different phylogenetic group clusters for core genome assignment. For visualization SVG files were generated with BRIG and in-house scripts.

Customized microarray design, analysis and quality control

The web based eArray application (Agilent Technologies, http://www.genomics.agilent.com/en/Custom-Design-Tools/eArray) was used for microarray design. Raw signal intensity values were determined using Agilent standard protocol for Affymetrix Microarray Analyzer from ATLAS-Biolabs. Probe level values (Agilent 'gProcessedSignal') were preprocessed using Quantile normalization from R-package preprocessCore (https://www.bioconductor.org/packages/devel/bioc/manuals/preprocessCore/man/preprocessCore.pdf) and log transformed.

Statistical analysis and Sliding Window calculation

Data/values of the treatment group (n = 3 samples, D5St1, D5St2, D5St4) and the control group (n = 4 samples, D5Wu1, D5Wu2, D5Wu3, D5Wu4) were averaged. Test-statistics were calculated using a two-sample t-test method with unequal variances (Welch-Test). The log-fold changes were calculated via the difference of the group means. The following two criteria were applied to estimate the significance of differential expression: (i) corrected T-test p-value < 0.05, multiple testing corrections was performed using fdr = false discovery rate (Benjamini Hochberg), and (ii) at least one of the two mean expression values (control and/or treatment) > 0.5. A low absolute fold change was no criterion for exclusion as long as the other criteria were fulfilled. Overrepresentation analyses of the candidate genes compared to the whole genome were performed in order to elucidate significant enriched gene sets by Gene Ontology (GO) terms using fishers exact test method (p-value < 0.05, not corrected). All statistical calculations were performed via in-house developed scripts using standard methods from R/Bioconducter platform (Gentleman et al., 2004) and appropriate packages. In order to determine genomic regions of *K. radicincitans* DSM 16656^T differentially expressed (up- or down-regulated) in response to root exudates, a genome-wide sliding window calculation was performed considering a window size of 15 consecutive genes.

Microbiome PhyloChip analysis

In high quality DNA the bacterial 16S rRNA genes were amplified using the degenerate forward primer: 27F.1 5'-AGRGTTTGATCMTGGCTCAG-3' and the non-degenerate reverse primer: 1492R.jgi 5'-GGTTACCTTGTTACGACTT-3'. Thirty-five cycles of bacterial 16S rRNA gene PCR amplification were performed. 16 samples (four replicates of root and shoot non-inoculated and inoculated by *K. radicincitans*) were moved forward for hybridization at Second Genome, Inc. (http://www.secondgenome.com/). For each sample, amplified products were concentrated using a solid-phase reversible immobilization method for the purification of PCR products and quantified by electrophoresis using an Agilent 2100 Bioanalyzer®. PhyloChip Control Mix™ was added to each amplified product. Bacterial 16S rRNA gene amplicons were fragmented, biotin labeled, and hybridized to the PhyloChip™ Array, version G3. PhyloChip arrays were washed, stained, and scanned using a GeneArray® scanner (Affymetrix). Each scan was captured using standard Affymetrix software (GeneChip® Microarray Analysis Suite). From each of the purified PCR products, 500 ng were

fragmented and hybridized. Assuming an average GC content of 54% (based Greengenes database of 16S rRNA genes) and an amplicon length of 1,465 bp, 3.3+E11 (330 billion) molecules were assayed from each sample. Second Genome's PhyloChip processing software, Sinfonietta, executes a multistage analysis (Probst et al., 2014).

References

Bloemberg, G.V., Wijfjes, A.H., Lamers, G.E., Stuurman, N., and Lugtenberg, B.J. (2000) Simultaneous imaging of *Pseudomonas fluorescens* WCS365 populations expressing three different autofluorescent proteins in the rhizosphere: new perspectives for studying microbial communities. *Mol Plant Microbe Interact* **13**: 1170-1176.

Fankem H, Tchuisseu TGV, Ngo NL, Nguesseu NG, Nwaga D & Etoa F-X (2014) Maize (Zea mays) growth promotion by rock-phosphate solubilising bacteria isolated from nutrient deficient soils of Cameroon. *African Journal of Microbiology Research* **8**: 3570-3579.

Gentleman, R.C., Carey, V.J., Bates, D.M., Bolstad, B., Dettling, M., Dudoit, S. et al. (2004) Bioconductor: open software development for computational biology and bioinformatics. *Genome Biol* **5**: R80.

Gerhardt, P., Murray, R., Krieg, N.R., and Wood, W.A. (1994) *Methods for general and molecular bacteriology*. Washington, DC: ASM Press.

Gonzales, M.F., Brooks, T., Pukatzki, S.U., and Provenzano, D. (2013) Rapid protocol for preparation of electrocompetent *Escherichia coli* and *Vibrio cholerae*. *J Vis Exp* **80**: 50684.

Koboldt, D.C., Zhang, Q., Larson, D.E., Shen, D., McLellan, M.D., Lin, L. et al. (2012) VarScan 2: somatic mutation and copy number alteration discovery in cancer by exome sequencing. *Genome Res* **22**: 568-576.

Lagendijk, E.L., Validov, S., Lamers, G.E., de Weert, S., and Bloemberg, G.V. (2010) Genetic tools for tagging Gram-negative bacteria with mCherry for visualization in vitro and in natural habitats, biofilm and pathogenicity studies. *FEMS Microbiol Lett* **305**: 81-90.

Li, H., and Durbin, R. (2009) Fast and accurate short read alignment with Burrows-Wheeler transform. *Bioinformatics* **25**: 1754-1760.

Nautiyal CS (1999) An efficient microbiological growth medium for screening phosphate solubilizing microorganisms. *FEMS Microbiol Lett* **170**: 265-270.

Probst, A.J., Birarda, G., Holman, H.Y., DeSantis, T.Z., Wanner, G., Andersen, G.L. et al. (2014) Coupling genetic and chemical microbiome profiling reveals heterogeneity of archaeome and bacteriome in subsurface biofilms that are dominated by the same archaeal species. *PLoS One* **9**: e99801.

Ruppel, S., Ruhlmann, J., and Merbach, W. (2006) Quantification and localization of bacteria in plant tissues using quantitative real-time PCR and online emission fingerprinting. *Plant Soil* **286**: 21-35.

Seemann, T. (2014) Prokka: rapid prokaryotic genome annotation. *Bioinformatics* **30**: 2068-2069.
